# A scaffold-degradable single-rivet ASD occluder with amplatzer-like design: a multicenter randomized controlled trial

**DOI:** 10.1007/s12928-026-01299-7

**Published:** 2026-06-19

**Authors:** Liqing Zhao, Jiawei Gao, Xin Zhou, Gangcheng Zhang, Yongsheng Gao, Tianli Zhao, Fengwei Zhang, Qiguang Wang, Jin Zhang, Pengjun Zhao, Jing Sun, Weiwei Zhou, Jian Zhang, Zhi Chen, Sun Chen, Kun Sun

**Affiliations:** 1 Department of Pediatric Cardiology Xinhua Hospital, Shanghai Jiao Tong University School of Medicine, Shanghai, China; 2 Department of Cardiovascular Medicine, Zhongnan Hospital of Wuhan University, Hubei, China; 3 Department of Cardiac Surgery, The First Hospital of Jilin University, Jilin, China; 4 Department of Cardiovascular Surgery, The Second Xiangya Hospital of Central South University, Hunan, China; 5 Department of Cardiovascular Surgery, Linyi People’s Hospital, Linyi, Shandong China; 6 Department of Congenital Heart Disease, General Hospital of Northern Theater Command, Liaoning, China; 7 Medical Department, Mallow Medical (Shanghai) Co., Ltd, Shanghai, China; 8 Department of Cardiology, Hunan Children’s Hospital, Changsha, Hunan China

**Keywords:** Atrial septal defect, Biodegradable device, Congenital heart defect, Catheterization closure

## Abstract

Metallic atrial septal defect (ASD) occluders are associated with various mid- to long-term complications. To address this, our team developed a novel biodegradable Pansy ASD occluder, and this multicenter RCT aimed to evaluate its effectiveness and safety. A prospective, multicenter, randomized controlled trial was carried out on adult and pediatric patients using the Pansy occluder with a biodegradable polydioxanone (PDO) framework. The primary efficacy endpoint of this clinical trial is the closure success rate at six months post-operation. From May 2021 to March 2023, a total of 127 patients were screened across seven research centers, with 112 patients (including 71 pediatric patients; 57 to the experimental group, 55 to the metal occluder control group) ultimately underwent occluder implantation. In these patients, the average defect size was 8.48 ± 2.86 mm in the experimental group and 10.41 ± 5.21 mm in the control group. The closure success rate at six months post-operation was 100% for both groups, and no residual shunt greater than 5 mm was detected at any follow-up time point. In the experimental group, one patient required device explantation at 10 months postoperatively due to abnormal tissue hyperplasia. Pathological analysis confirmed near-complete degradation of the PDO framework with smooth endothelial coverage. A total of 109 patients completed the 12-month post-operative follow-up, with both groups again demonstrating a closure success rate of 100%. The Pansy occluder shows promising safety and efficacy outcomes at 12 months, though longer-term data are needed to confirm durability.

**Graphical abstract:**

**Figure:**
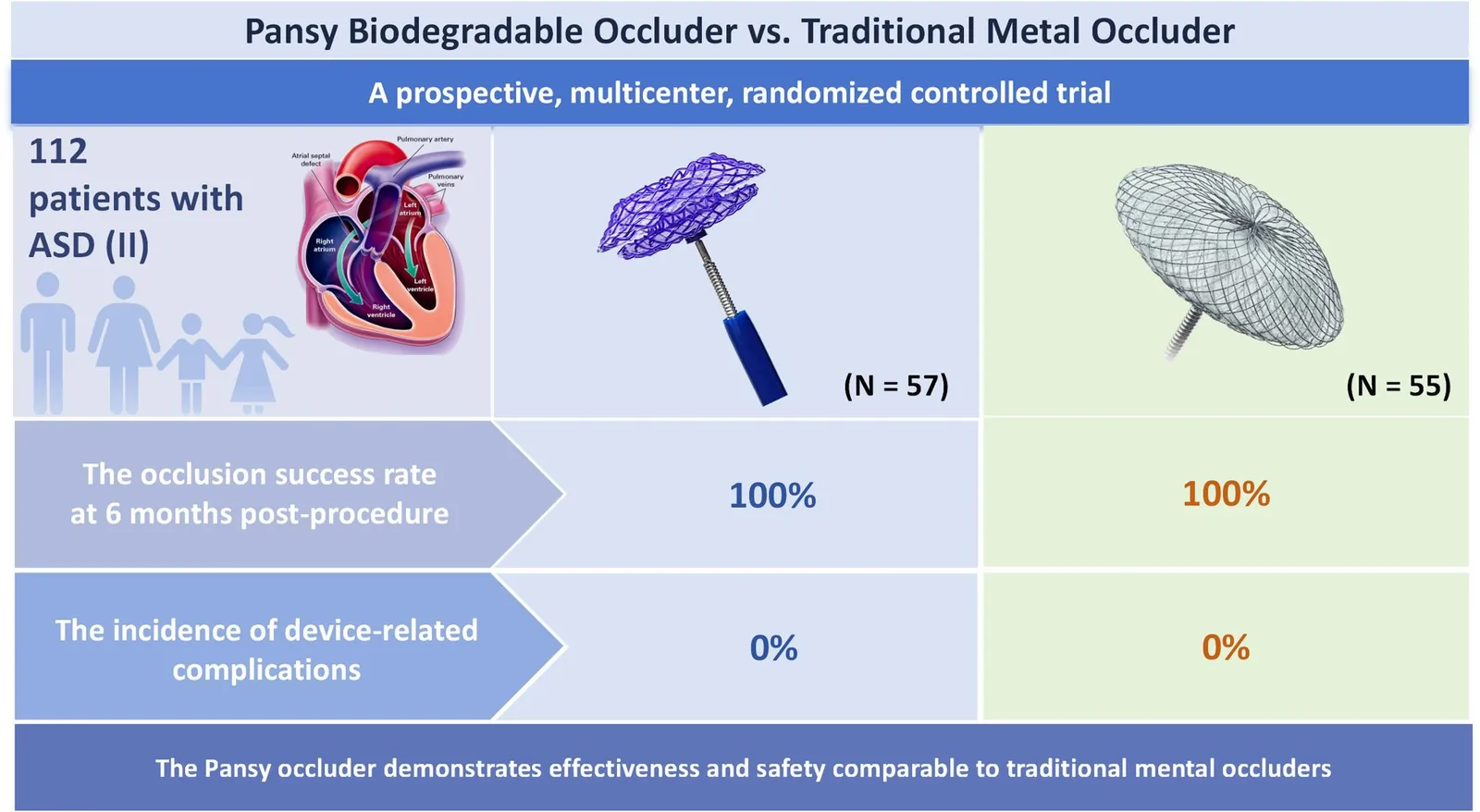

**Supplementary Information:**

The online version contains supplementary material available at https://doi.org/10.1007/s12928-026-01299-7.

## Introduction

Atrial septal defect (ASD) is a common congenital heart defect, with a reported incidence of approximately 1 per 1,000 live births [1]. Currently, nitinol-based occluders represent the primary clinical treatment. However, these devices carry inherent limitations, including nickel releasen and compression effects, which can lead to serious complications such as allergic reactions, cardiac perforation, and complete atrioventricular block [2–4]. Furthermore, long-term incidences of concurrent atrial fibrillation and mitral regurgitation can be as high as 20% [5, 6]. These subsequent complications are often managed via transcatheter interventions, including radiofrequency ablation, left atrial appendage closure, and transcatheter mitral valve edge-to-edge repair (TEER). Critically, all such transcatheter interventions require transseptal puncture via the femoral vein to access the left atrium. Performing transseptal puncture, however, poses significant challenges in patients with a pre-existing ASD occluder in place [7].

To address the limitations of non-degradable devices, the development of biodegradable occluders has emerged as a promising alternative. In previous studies, partially biodegradable occluders (e.g., BioSTAR, BioDisk, and Double BioDisk) and fully biodegradable occluders (e.g., Chinese Lantern occluder, Double Umbrella occluder, and PCL-PLGA/Collagen occluder) have garnered significant attention. However, these devices still encounter challenges, including uneven endothelialization, a high rate of residual shunting, and transient postoperative atrial arrhythmias [8–11]. Furthermore, the elastic recovery properties of biodegradable polymer materials are significantly inferior to those of traditional alloy materials [12], resulting in reduced reliability and stability of the device after implantation. To enhance the sealing performance of these devices, various locking system designs have been implemented. For instance, the Absnow™ device employs a handle-locking mechanism [13], while the Chinese Lantern occluder utilizes a deployment line to achieve a “pull-fold” mechanism [11]. However, these designs may induce turbulent flow around the occluder, leading to platelet aggregation and activation, which can promote the formation of microthrombi [14]. Additionally, these mechanisms increase deployment complexity, thereby prolonging the learning curve for operators.

To address the aforementioned challenges, we designed a novel metal-free occluder composed of a polydioxanone (PDO) mesh framework, a polyethylene terephthalate (PET) membrane, and polyamide (PA) sutures, which we have named “Pansy”. The device is manufactured using a patented process that imparts superelasticity and self-expanding properties to the PDO mesh support structure. This innovation mitigates the disadvantage of high residual shunt associated with biodegradable occluders and enables a delivery and release process similar to that of metal occluders. This study is a prospective, multicenter, randomized controlled trial conducted in China, encompassing a wide age range from pediatric to adult patients, aimed at validating the efficacy and safety of this occluder as non-inferior to traditional occluders.

## Materials and methods

The data will not be made available to other researchers for the purposes of reproducing the results or replicating the procedure.

### Device design

**Fig. 1:**
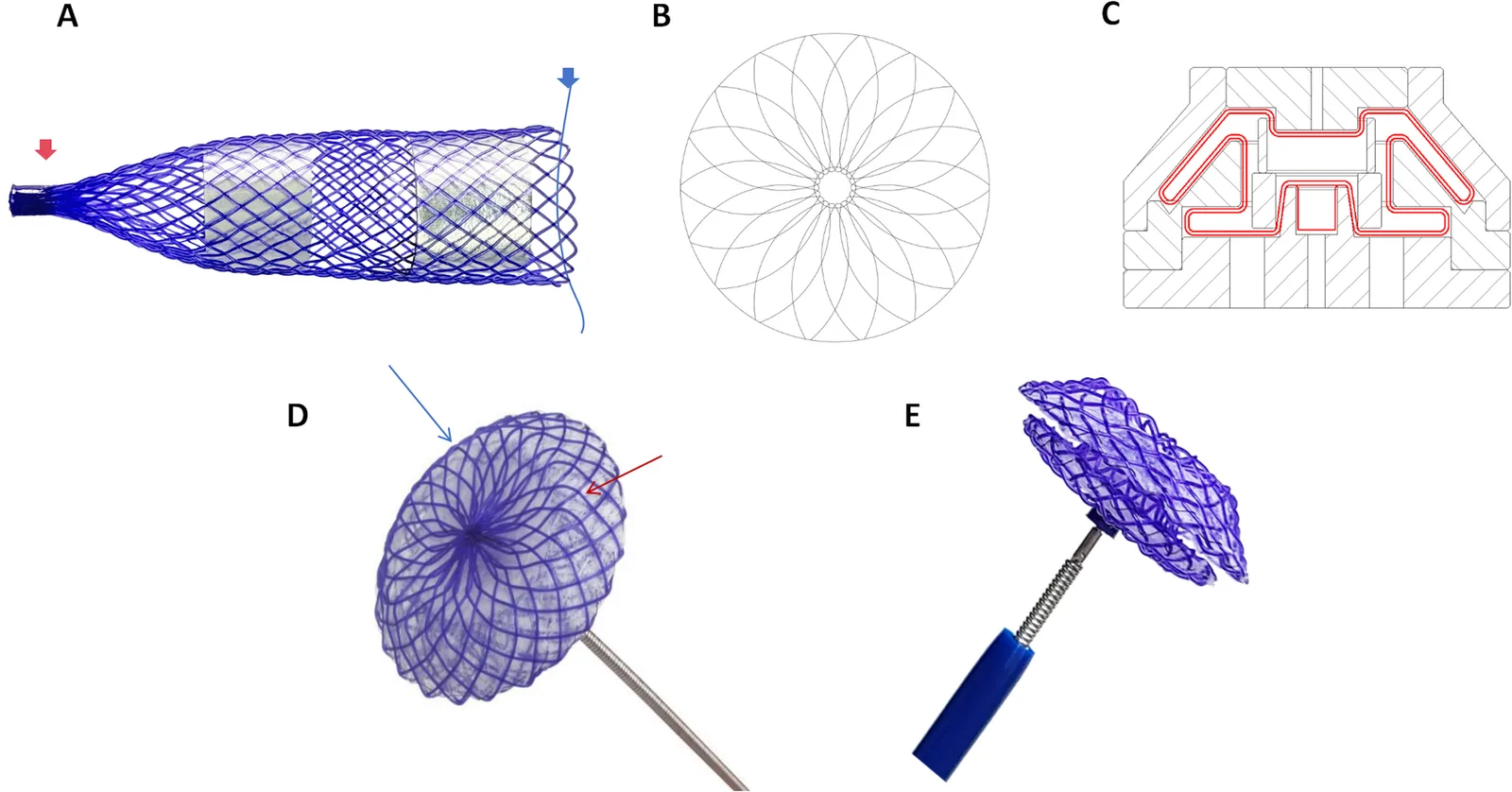
The manufacturing process of the Pansy occluder. **A**. The integrated tubular network. **B**. Schematic diagram of the left side of the disc. **C**. Schematic diagram of the custom mold, with the red outline indicating the position of the device. **D**. Left side view of the device (the “blue thin arrow” indicates the biodegradable PDO structural framework; the “red thin arrow” designates the non-degradable PET membrane). **E**. Side view of the device, showing the connector attached to the transport cable via threads. The “bold red arrow” points to the right side of the disc, which is shaped through a hot melting process and features internal threads that form a connector. The “bold blue arrow” points to the left side of the disc, which is finished with seams to create a smooth surface

Previous research initiated in 2006 established the conceptual foundation for an ideal degradable atrial septal occluder. Based on this foundation, the Pansy occluder was developed and manufactured for the present study. This device comprises a mesh framework, connector, membrane, and sutures. The mesh framework and connector are woven from PDO monofilament or multifilament, while the membrane is made of PET, and the sutures are made of PA (Fig. 1D, E). The PDO filaments, treated through a specialized process, exhibit a controllable degradation rate. Preliminary trials have shown that the degradation process aligns with the requirements for defect repair, with the final hydrolysis products being carbon dioxide and water. During the initial three months, the framework maintains its full mechanical strength, providing the primary closure force while facilitating tissue ingrowth into the non-degradable PET occlusion membrane. Within 12 months, near-complete release of the primary degradation product, 2-hydroxyethoxyacetic acid (HEAA), is achieved (Supplementary Material S1). The proprietary manufacturing process and custom molds (Fig. 1C) endow the mesh framework with unique superelasticity and self-expansion properties, making it the first known self-expanding degradable occluder. The device exhibits excellent shaping effects, sufficient gripping strength, and outstanding fatigue resistance (Supplementary Material S2). The left disc surface of the PDO woven mesh tube features a continuous and smooth mesh without exposed filament ends. By introducing a closing line and tightening it into a ring, a smooth distal disc without rivets is formed (Fig. 1B). The mesh framework and connector are integrated, with the right side of the disc retaining a sufficient length of biodegradable filament for thermal melting and subsequent cooling solidification to form an internal threaded connector (Fig. 1A), which can be connected or disconnected via the threads with the delivery cable. Consequently, the operational procedure for the biodegradable occluder during surgery is nearly identical to that of traditional metal occluders, requiring minimal additional training for operators. The occluder is available in various sizes, with waist diameters ranging from 4 mm to 34 mm, and can be delivered through 8 F to 12 F sheaths.

### Study design and oversight

This study is a multicenter, prospective, randomized controlled trial designed to evaluate the efficacy and safety of the Pansy biodegradable occluder in comparison to traditional metal occluders. The trial is being conducted at seven cardiac centers in China (Registration No.: ChiCTR1900027414).

The study design complies with the regulatory requirements outlined in the Clinical Trial Quality Management Guidelines for Medical Devices. The trial protocol has been approved by the National Medical Products Administration (NMPA) (Supplementary Material S3) and by the ethics committees of each participating center (Leading Unit Approval No.: XHEC-B-2019-006) (Supplementary Material S4). The sponsor is responsible for quality control of the trial, selection and management of trial centers, data management and analysis, as well as communication with the regulatory authorities to ensure compliance with the trial results and data accuracy. All adult participants (≥ 18 years old) signed a written informed consent form themselves. For minors under 8 years old, informed consent was obtained from their guardians, who signed the written informed consent form on behalf of the minors. For minors aged 8 years and older, if they possess the ability to write, both the minors and their guardians signed the written informed consent form together (Supplementary Material S5).

### Patient, randomization and follow up

The inclusion criteria are as follows: (1) Age ≥ 2 years; (2) A confirmed diagnosis of secundum ASD with a defect diameter ranging from 5 to 36 mm, accompanied by evidence of right ventricular volume overload; (3) A small defect with intermittent right-to-left shunting and suspected embolic sequelae; (4) A minimum distance of 5 mm between the defect margin and major cardiac structures (e.g., venae cavae, coronary sinus, pulmonary veins), and at least 7 mm from the atrioventricular valves; (5) The diameter of the atrial septum must exceed the diameter of the left disc of the selected occluder; (6) All participants must voluntarily agree to participate and sign an informed consent form. Compliance with either the second or third criterion is sufficient.

The main exclusion criteria include the presence of additional cardiac anomalies that require surgical correction, severe pulmonary hypertension, active infective endocarditis or bleeding disorders, a systemic infection within the past month, an inability to take aspirin or other anticoagulant therapy for six months, and the presence of intracardiac thrombus.

**Fig. 2:**
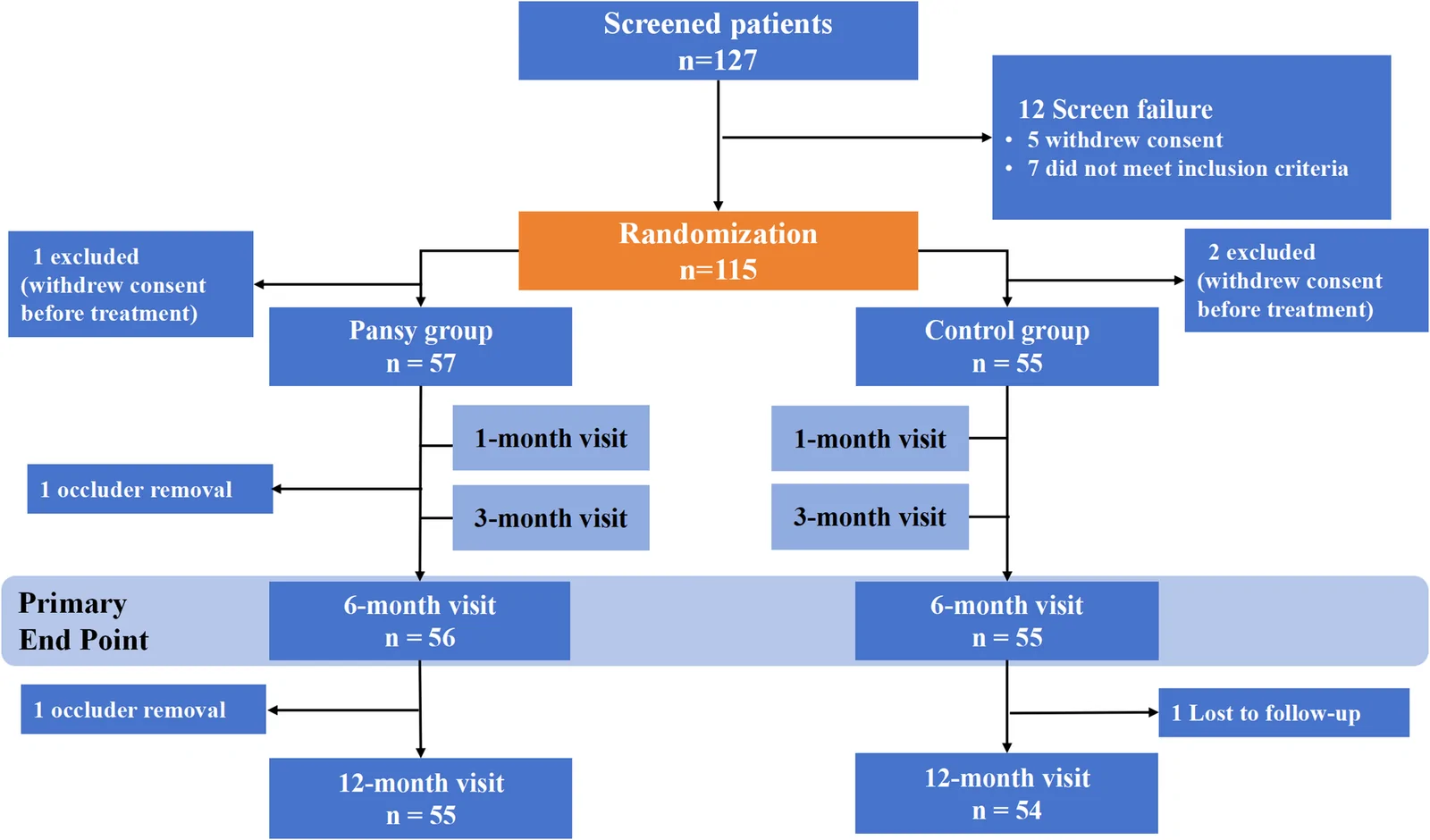
Flowchart of the study

Patients were randomized in a 1:1 ratio by center using a blocked randomization scheme. Random sequences were generated by a centralized randomization system, and allocation concealment was ensured using sealed opaque, sequentially-numbered envelopes. Follow-up assessments were scheduled at discharge, 1 month, 3 months, 6 months, and 12 months post-procedure (Fig. 2). The primary efficacy endpoint was assessed at 6 months postoperatively, with follow-up was conducted until 12 months postoperatively. Follow-up evaluations included physical examinations, electrocardiography (ECG), and either transthoracic echocardiography (TTE) or transesophageal echocardiography (TEE). Complications, adverse events(AEs), and medication usage were documented at each follow-up visit. Detailed trial procedures and follow-up plans are provided in Supplementary Material S6.

### Procedure

All procedures were performed by physicians qualified in percutaneous transcatheter ASD closure.The procedure was performed via the femoral vein under TTE or TEE guidance, with intravenous heparin administered at a dosage of 100 U/kg during the operation. The size of the device was selected based on the maximum defect diameter measured by TTE or TEE. Adult patients typically received a device with a waist diameter 4 to 6 mm larger than the defect, and pediatric patients received a device 2 to 4 mm larger.

The delivery and implantation method of the Pansy bioabsorbable device was similar to that of traditional metallic occluders. The device’s release was monitored using both digital subtraction angiography(DSA) and ultrasound. Following implantation, aspirin was administered as antithrombotic therapy at a dosage of 3–5 mg/kg per day for a minimum duration of six months. The postoperative anticoagulation regimen could be adjusted by the researchers based on individual patient characteristics.

### Study end points

“Technical success” is defined as the successful deployment and implantation of the device in the correct position during the index procedure, as confirmed by TTE or TEE. The delivery system was smoothly advanced, easily operated, accurately positioned, and withdrawn without difficulty.

The primary efficacy endpoint is the occlusion success rate at 6 months post-procedure. Successful occlusion is defined as the effective deployment and implantation of the device at the target site, confirmed by either TTE or TEE. Echocardiography confirmed correct device position, normal morphology, and the absence of or only minimal to mild residual shunting, with no associated clinical manifestations or complications. Minimal residual shunt, as assessed by color Doppler for left-to-right flow, is defined as a left-to-right shunt signal with a diameter of < 5 mm and may be left untreated [15].

The secondary endpoint is the incidence of device-related complications occurring during the procedure, throughout the postoperative period until discharge, and at each follow-up visit. Common complications include: (1) Residual shunt ≥ 5.0 mm (moderate residual shunt and above); (2) Thromboembolism; (3) Gas embolism; (4) Perinatal or long-term headache/migraine; (5) Hematoma and femoral arteriovenous fistula at the puncture site; (6) Pericardial tampon; (7) Displacement and fall off of the occluder; (8) Arrhythmia related to the occluder; (9) Aortic to the right atrium and left atrium fistula; (10) Hemolysis; 11) Other rare complications.

### Adverse event assessment

All adverse events (AEs) and serious adverse events (SAEs) were meticulously recorded from the time of surgery until discharge, as well as at each follow-up visit. Each event was assessed by the investigator and classified as definitely related, possibly related, possibly unrelated, or indeterminate in relation to the investigational device. Any SAEs associated with or potentially linked to the experimental device were reported immediately to the drug supervision department of the province, autonomous region, or municipality directly under the central government where the sponsor was located. Simultaneously, reports were submitted to the drug supervision department and the health management department of the province, autonomous region, or municipality where the medical device clinical trial institution was situated for further evaluation.

### Statistical analysis

This study employed a non-inferiority design with an active control, referencing previous literature and data from similar clinical trials, where the occlusion success rate of nitinol occluders ranges from 98% to 100% [16, 17]. Based on an alpha level of 0.025 (one-sided test), a beta of 0.2 (power of 80%), and a non-inferiority margin (δ) of -10%, the sample size was calculated using a large-sample normal approximation method. Participants in the trial group and the control group were randomly assigned in a 1:1 ratio. Using PASS 14 software, the calculation determined that a minimum of 32 eligible patients per group was required. Accounting for potential dropout rates, each group was planned to enroll 56 patients, resulting in a total of 112 participants.

Normality of data distribution was assessed using the Shapiro-Wilk test. All continuous data were normally distributed and thus expressed as mean ± standard deviation (SD). Clinically relevant continuous variables were supplemented with range values where appropriate. Categorical variables will be expressed as counts (percentages). Student’s t-test and chi-square test will be employed to assess statistical differences between means and proportions, respectively. All significance tests will be two-sided, with P-values < 0.05 considered statistically significant. All statistical analyses will be performed using SPSS Statistics software (version 26.0).

## Result

### Baseline characteristics

Between May 2021 and March 2023, a total of 127 patients were screened across seven centers. Twelve patients were excluded for failing to meet the inclusion criteria, and three withdrew their informed consent before the implantation of an occluder, resulting in 112 patients who finally underwent the implantation procedure. Of these, 57 patients were assigned to the biodegradable occluder group, while 55 patients were assigned to the control group. Baseline demographic and clinical characteristics are summarized in Table 1. Notably, the study population included 71 pediatric patients (aged < 18 years), with 41 (57.7%) in the experimental group and 30 (42.3%) in the control group. Among the patients who met inclusion criteria 2, the mean baseline defect size in the experimental group was 8.48 ± 2.86 mm (range: 5.0–17.0 mm), and the mean size of the selected occluder was 13.62 ± 3.99 mm. In the control group, the mean baseline defect size was 10.41 ± 5.21 mm (range: 5.0–26.0 mm), and the mean size of the selected occluder was 15.88 ± 6.64 mm. Among the patients who met inclusion criteria 3, there were 17 cases: 10 in the experimental group and 7 in the control group. These patients had a history of transient ischemic attacks (15 cases), bilateral posterior horn ischemia (1 case), or bilateral multiple lacunar infarcts and ischemic foci (1 case), all of whom tested positive on the bubble test at enrollment.

**Table 1:** Baseline characteristics

Characteristics	Experimental Group (*n* = 57)	Control Group (*n* = 55)	*P*-value
Age, yrs	18.35 ±15.95	20.42 ±16.81	0.506
<18 yrs, n (%)	41(71.9)	30(54.5)	0.087
Female, n (%)	31 ( 54.4)	40 ( 72.7)	0.044
Weight(kg)	42.79 ±21.66	43.05 ±24.79	0.952
Height(cm)	143.00 ±26.78	139.07 ±30.33	0.469
The size of defect, mm	7.26 ±3.73	9.34 ±5.64	0.002
**Device information**			
Device size in waist, mm	11.75 ±4.88	14.91 ±6.85	0.006
Device-to-defect ratio	1.98 ± 1.08	2.00 ± 1.29	0.9
**Patients meeting inclusion criterion 2**	47	48	/
The size of defect, mm	8.48 ± 2.86	10.41 ± 5.21	0.028
**Patients meeting inclusion criterion 3**	10	7	/
Transient ischemic attacks(TIA)	8	7	/
Bilateral ischemia of the posterior horns of the lateral ventricles	1	0	/
Bilateral multiple lacunar infarctions and ischemic foci	1	0	/

Notes: Values are mean ± SD, or n, or n(%)

### Follow-up results

#### Efficacy endpoints

During the follow-up period, one patient in the experimental group underwent device removal due to infective endocarditis and subsequently withdrew from the trial. Consequently, 111 patients were included in the Per-Protocol Set (PPS) analysis. The primary efficacy endpoint was the occlusion success rate at 6 months post-procedure. In the PPS analysis, which included 111 patients, the 6-month occlusion success rate reached 100% in both group (Table 2).

**Table 2:** Primary efficacy endpoint and complications

Primary effcacy endpoint	Experimental Group	Control Group
	*n* = 56	*n* = 55
Success rate at 6 months	56(100)	55(100)
**12-month-follow up**	*n* = 55	*n* = 54
Success rate at 12 months	55 (100)	54 (100)

A total of 109 patients completed the 12-month postoperative follow-up, and the occlusion success rate was maintained at 100% in both groups.

#### Safety endpoints

The incidence of complications (as defined in the methods section) in this study was recorded intraoperatively, postoperatively until discharge, and at each follow-up visit. The analysis showed that the complication rate was 0% at all follow-up time points in both groups (Table 3). No serious device-related complications, including mid~large residual shunts and new arrhythmias, were observed in either group. Of the 17 patients who met inclusion criteria 3, there was significant clinical improvement at 6-month follow-up, with no new cerebral infarcts or ischemic lesions observed.

**Table 3:** Incidence of adverse events during 12-months follow up

Indicator	Experimental Group (*n* = 57)	Control Group (*n* = 55)	*P*-value
**Adverse Events**	32 (56.1)	37 (67.3)	0.225
**Device-Related**	0 (0)	1 (1.8)	1
**Serious Adverse Events**	5 (8.8)	5 (9.1)	1
**Device-Related**	0 (0)	0 (0)	1

Notes: Values are n (%)

Among the 109 patients who completed the 12-month postoperative follow-up, a total of 69 patients reported 147 AEs, for which sufficient follow-up data were obtained. 11 patients experienced headache events, with 7 cases occurring in the trial group and 4 in the control group. Only 1 patient in the control group experienced transient frequent headaches 1 month postoperatively, raising the possibility of a microthrombus as a potential cause of the symptoms. The headache completely resolved after the addition of clopidogrel in combination with routine aspirin treatment. The remaining 10 patients had infrequent headaches (< 3 episodes/year) or clearly identified causes for their headaches, which the investigator deemed unrelated or possibly unrelated to the investigational device.

The details of the SAEs are provided in the supplementary Material S7. During the follow-up period, 11 SAEs occurred in 10 patients, with 5 patients in the trial group and 5 in the control group (*P* = 1). Device explantation was required in two trial group cases.

One patient was hospitalized 1 month after surgery due to recurrent fever and was diagnosed with a bloodstream infection (infective endocarditis) caused by Staphylococcus aureus. The patient underwent resection of the vegetation, removal of the occluder, and surgical repair of the ASD. Microbial testing conducted by a third-party laboratory on samples from the same batch of occluders did not detect any microbial contamination. A total of 22 patients were implanted with devices from the same batch across four centers, and no similar infection events were reported in the other 21 cases. Consequently, the researchers concluded that this serious adverse event is unrelated to the trial device.

Another pediatric patient showed normal occluder position but mobile tissue (6.2 × 11.0 mm) in the left atrium during 10-month follow-up. After multidisciplinary review, the device was explanted. Based on the analysis of the specimen retrieved from the patient’s left atrium and the corresponding pathological findings (Fig. 4 and Supplementary Material S8), the outline of the right disc of the implanted biodegradable occluder is visible. The mesh structure of the device’s right side has mostly degraded, with smooth and even tissue overgrowth covering it. The mesh structure on the left side has completely degraded; however, the tissue overgrowth there is abnormal, presenting as a cord-like structure attached to the interatrial septum with a bulbous end. The foreign body removed from the left atrium during surgery is likely related to the proliferation and abnormal growth of granulation tissue. This indicates that during the degradation process, the newly formed tissue has successfully achieved coverage of the occlusion site, without any component protrusion due to “incomplete tissue coverage.” The abnormal tissue was diagnosed as excessive fibrous tissue hyperplasia, and no similar tissue hyperplasia events occurred in the remaining 111 patients. We believe this more likely reflects an idiosyncratic healing response, analogous to aberrant scar formation, rather than a systemic inflammatory issue triggered by PDO degradation. Such a reaction could theoretically occur with any implant and has no direct causal relationship with the device’s biodegradable nature.

**Fig. 3:**
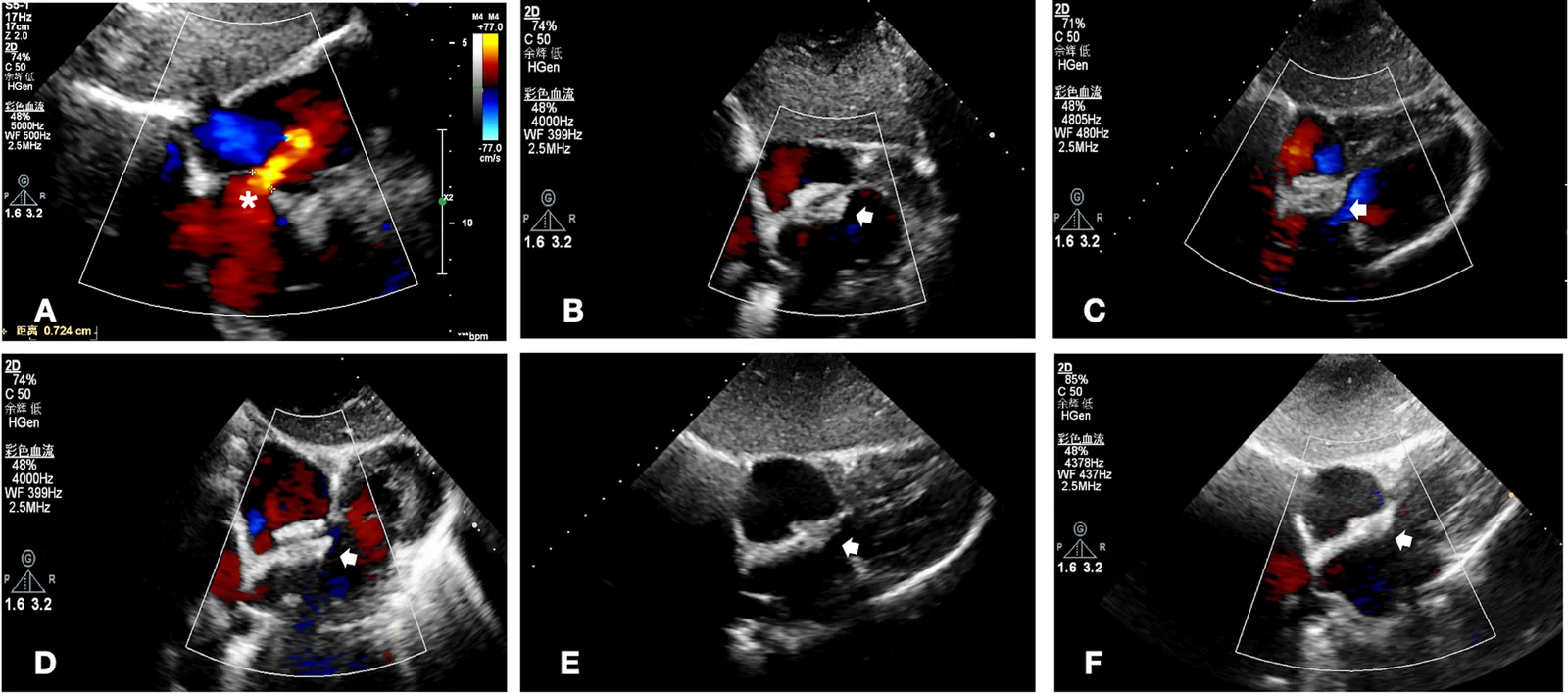
Echocardiograms (subxiphoid view) of the same patient in the experimental group, illustrating the progression from the screening period to the 12-month post-surgery follow-up. **A**. Screening period, with the “white asterisk” indicating the presence of an atrial septal defect; **B**. Pre-discharge re-examination following surgery; **C**. One month post-surgery follow-up, demonstrating clear profiles of the occluder’s dual discs in the correct position, with no residual shunt; **D**. Three months post-surgery follow-up; **E**. Six months post-surgery follow-up; **F**. Twelve months post-surgery follow-up. Note: The “white arrow” indicates the position of the occluder

**Fig. 4:**
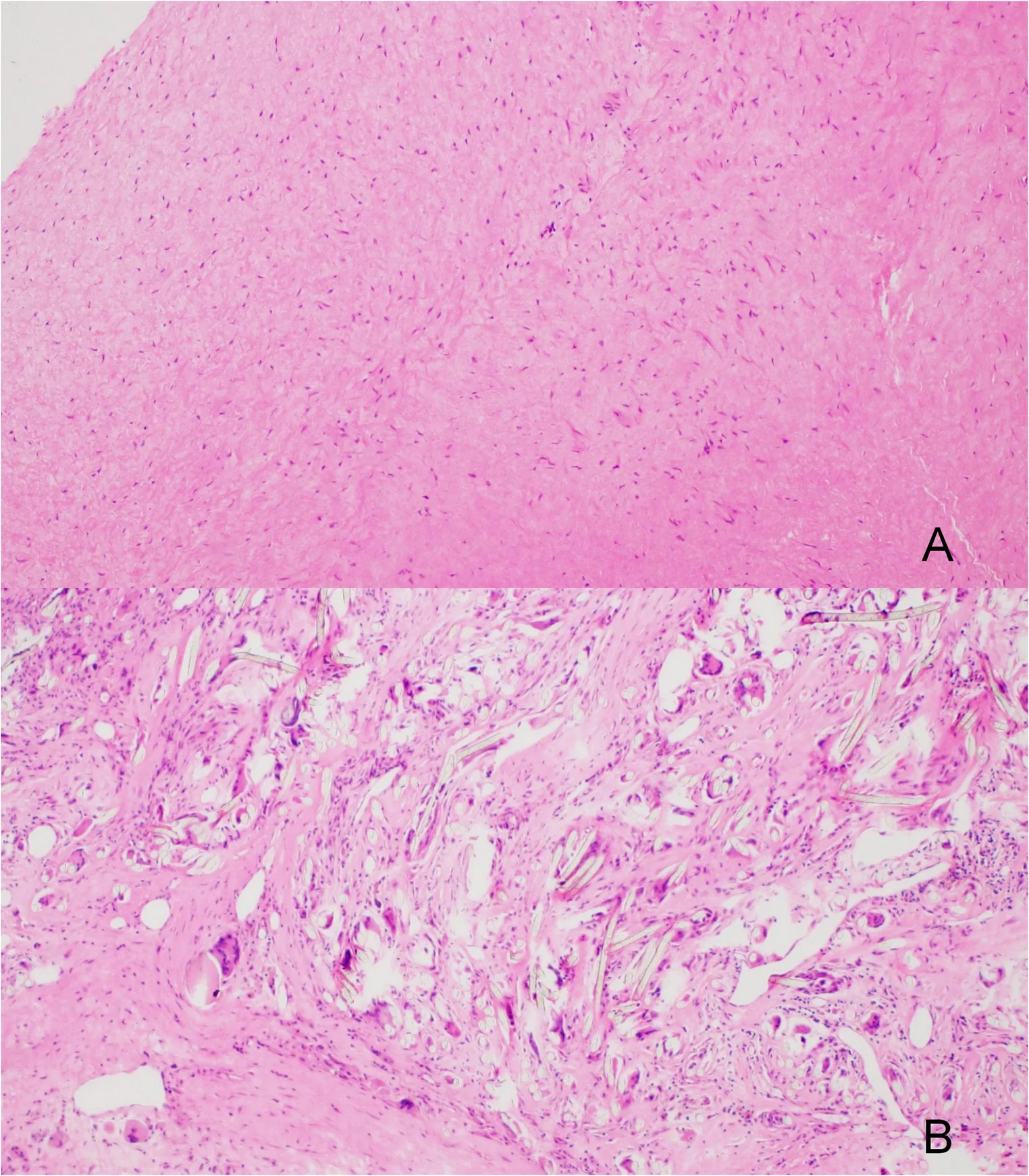
Histopathological analysis of the explanted hyperplastic tissue from the left atrium. **A**. Low-magnification view showing the fibrous strand (arrow) attached to the interatrial septum (hematoxylin and eosin [HE] staining, ×40). **B**. High-magnification view of the bulbous end, demonstrating its composition and chronic inflammatory cell infiltrates (HE staining, ×100). Pathological examination confirmed that the tissue represents abnormal fibrous hyperplasia, unrelated to the biodegradation process of the device

Other SAEs (fractures, thyroid malignancy, early pregnancy termination, fevers) requiring hospitalization or diagnostic evaluation were all deemed unrelated to the occluder.

### Residual shunting

At 6-month follow-up, 8 patients had minor residual shunts, all in the test group, and none of the remaining patients had moderate to large residual shunts (residual shunts > 5 mm). The ultrasound reports all showed normal position and normal shape of the occluder. The residual shunts observed in these patients did not significantly impact clinical symptoms, and all patients maintained normal chamber sizes without hemodynamic significance. No complications related to residual shunting were reported during the follow-up period. Among the eight patients, only two presented with a residual shunt exceeding 2 mm. At the 12-month follow-up, complete resolution was observed in one patient, whereas the shunt size remained stable in the other.

### Degradation of the bioabsorbable occluder

Currently, there is no precise method for evaluating the degradation of degradable occluders within the human body. Assessments primarily rely on ultrasound images and the expertise of ultrasound physicians, who make judgments based on morphological observations. In this study, echocardiography was employed to qualitatively assess the degree of degradation of degradable occluders, utilizing the experience of ultrasound specialists. In the subxiphoid four-chamber view of the same participant, at 3 months post-operation and earlier, the biodegradable occluder appeared as a hyperechoic region protruding from the surrounding endocardium, with relatively clear visibility of the device’s edges. At the 6-month follow-up, the hyperechoic region had merged with the surrounding endocardium, and the edges of the occluder became less distinct. By the 12-month follow-up, echocardiography did not detect any identifiable hyperechoic region (Fig. 4).

## Discussion

This study reports the initial clinical evaluation of a novel biodegradable Pansy ASD occluder. Constructed primarily from poly-p-dioxanone (PDO), this device represents an innovative approach as a potentially self-expanding biodegradable ASD occluder and is offered in various sizes suitable for both adults and children. This trial constitutes the first prospective, multicenter, randomized controlled clinical study evaluating this device type and included both pediatric and adult patients. Our 12-month follow-up data demonstrate that the Pansy occluder achieved non-inferiority compared to conventional nitinol occluders in terms of efficacy and safety during the short-term postoperative period.

Our study demonstrated that both the Pansy occluder and the conventional metal occluder achieved a 100% closure success rate at 6 months post-procedure, with no residual shunts greater than 5 mm detected at any follow-up time points (1, 3, 6, and 12 months). These findings indicate the non-inferiority of the Pansy occluder in terms of efficacy compared to traditional metal occluders. Among 71 pediatric patients (under 18 years of age), the Pansy occluder exhibited similar efficacy and safety outcomes as those observed in adult patients. Due to the complete degradability of PDO material, this occluder mitigates the potential long-term complications associated with traditional metal occluders, such as metal hypersensitivity and chronic inflammation, which may offer significant advantages, particularly for pediatric patients.

Interventional techniques represent a significant advancement in the field of cardiovascular medicine. As one of the most commonly used implantable devices, atrial septal occluders should ideally facilitate the heart’s self-repair process while minimizing the residual presence of foreign material to reduce the risk of long-term complications. However, the prolonged use of conventional metal occluders has been closely associated with complications such as tissue compression [18–20]. Although biodegradable occluders have been proposed to mitigate these issues, they still face challenges, including mismatched rates of degradation and endothelialization, incomplete degradation, and poor elastic recovery requiring complex locking mechanisms [21–23]. In this study, a patented self-expanding bioabsorbable occluder frame has been designed to solve the problem of poor elastic recovery of “locker” biodegradable occluders, thus avoiding arrhythmias caused by excessive mechanical stimulation of tissues due to forceful “locking”. Additionally, the PET membrane does not hinder future transseptal puncture procedures, offering possibility for potential subsequent interventional treatments.

Residual shunting remains one of the major challenges associated with previous biodegradable occluders, primarily due to the decline in occlusion effectiveness following the initiation of the degradation process, which significantly impacts the long-term efficacy of biodegradable devices [24, 25]. For biodegradable occluders, premature initiation of degradation before complete tissue healing may lead to device collapse, fragmentation, and thrombus formation. Conversely, if the degradation process is excessively prolonged, it may induce adverse inflammatory responses, thereby hindering the formation of healthy neotissue. These limitations not only contribute to residual shunting but may also increase the risk of complications such as thromboembolism, arrhythmias, and myocardial scarring. Therefore, compared to metal occluders, biodegradable occluders may present certain disadvantages in terms of the long-term efficacy of ASD closure [13, 21].

Achieving a balance between the degradation process and the tissue healing response is crucial for minimizing postoperative residual shunting in biodegradable occluders. Several studies have indicated that complete coverage of the central membrane by fibrous granulation tissue requires approximately 1 to 3 months, whereas endothelialization of the occluder discs, particularly the central bulging portion, takes around 3 to 6 months [26]. Consequently, the occluder is no longer needed after six months post-procedure. The Pansy occluder, designed with a degradable framework combined with a non-degradable PET occlusion membrane, integrates the advantages of structural support, degradation timeline, and material biocompatibility. The degradable PDO material hydrolyzes into non-toxic byproducts such as oxalate and glycine, with a degradation rate well-matched to the tissue healing process [27, 28]. Furthermore, the inclusion of the PET occlusion membrane enhances short-term closure efficacy while providing stable support for tissue healing, thereby reducing the incidence of moderate or severe residual shunting.

In contrast, the Biostar occluder reported a residual shunting rate as high as 30% (21.8% mild, 9.1% moderate) within two years post-procedure, with no clearly identified predictive factors, indicating certain limitations in its closure performance [29]. The Absnow™ occluder, a fully biodegradable self-expanding double-disc device made from poly-L-lactic acid (PLLA), showed new-onset residual shunting in 3 out of 5 patients during long-term follow-up in the first-in-human clinical trial, including 2 cases of moderate shunting and 1 case with right ventricular enlargement. These findings suggest limitations in the long-term closure effectiveness of the Absnow™ device [21], likely attributable to PLLA biodegradable material and the high acidity of PLLA degradation byproducts. We observed 8 cases of minor residual shunts (< 5 mm) in the test group, while none were detected in the control group. All shunts were deemed hemodynamically insignificantand caused no clinical symptoms or complications throughout follow-up. Compared to earlier biodegradable occluders (e.g., Biostar™ with a 30% total residual shunt rate, including 9.1% moderate/severe; Absnow™ with 40% moderate/severe shunt rate), the Pansy occluder demonstrates advantages in closure efficacy. We propose that this phenomenon relates to the dynamic “degradation-healing interplay” unique to biodegradable devices. In the initial months post-implantation, as the PDO framework begins hydrolytic degradation, subtle changes in mechanical support occur while tissue integration progresses. This transient asynchrony between degradation and tissue remodeling may permit minor early-stage shunting.

Due to the typically lower elasticity of biodegradable materials compared to nitinol alloys [21], one of the major challenges in developing bioabsorbable occluders is ensuring that the device reliably regains a stable double-disc shape after deployment. Various locking system designs have been introduced to enhance the sealing performance of these devices. However, these designs may generate turbulent blood flow around the occluder, promoting platelet aggregation and activation, which can lead to microthrombus formation [24]. In addtition, device-induced compression stress and stimulus responses will often cause atrial arrhythmias [30].The Pansy occluder, through a specialized manufacturing process, is endowed with unique elasticity and self-expanding properties, thereby eliminating the need for additional fixation mechanisms. Preliminary testing has demonstrated the device’s excellent shape retention, good self-adapting, sufficient clamping strength, and superior fatigue resistance (Supplementary Material S2). During the one-year follow-up of this trial, no device-related new-onset arrhythmias occurred in any of the patients and both the left and right discs remained securely apposed to the margins of ASD, with no observed structural instability or sealing defects. Moreover, the integrated structural design allows the delivery and deployment process to be identical to that of traditional metal occluders, significantly reducing the learning curve. In this trial, all occluder implantations were successfully completed on the first attempt. Additionally, if a size adjustment or device retrieval is required during the procedure, the occluder can be easily recaptured into the sheath for seamless removal and replacement.

The Pansy occluder features a patented single-rivet structure, providing a smooth occluder disc surface after implantation. This innovative design helps reduce the risk of thrombus formation caused by surface turbulence and minimizes mechanical damage to surrounding tissues during the procedure. The left disc of the PDO woven mesh tube is characterized by a continuous and smooth grid structure without exposed wire ends. By introducing a closure loop and tightening it into a ring, a rivet-free, smooth distal disc surface is achieved. This rivet-free design reduces the risk of thrombus formation, and no thrombi were observed in either group of patients during the follow-up period of this trial. The right disc adopts an integrated manufacturing process, the screw and nut structure provide sufficient tension.

### Limitation

Inevitably, there are several potential limitations in the present study. First, it is essential to recognize that the study enrolled a relatively limited number of patients (*n* = 112). Larger trials with extended follow-up are necessary to comprehensively evaluate long-term performance and rare complication risks. Second, this trial did not include patients with complex ASD with poor rim conditions, and the applicability of the device in such challenging cases requires further exploration and clinical experience. Additionally, a current limitation of all biodegradable occluders is their lack of radiopacity under DSA. Although no reports of device embolization have been noted in previous studies, if such an event occurs in peripheral vessels, there is currently no standardized management protocol. Therefore, for clinical application, careful patient selection is recommended, prioritizing cases with favorable anatomical conditions and conducting thorough pre-procedural assessments of defect location and morphology to minimize potential risks.

## Conclusions

The 12-month follow-up data demonstrate that the Pansy occluder is non-inferior to conventional nitinol occluders in terms of efficacy and safety during the short-term postoperative period.While no device-related serious adverse events (e.g., thromboembolism, cardiac perforation, or mortality) occurred in the 12-month follow-up, the single explantation case and higher incidence of minor residual shunts warrant continued surveillance.

## Supplementary Information

Below is the link to the electronic supplementary material.

Supplementary Material 1

Supplementary Material 2

Supplementary Material 3

Supplementary Material 4

Supplementary Material 5

Supplementary Material 6

Supplementary Material 7

Supplementary Material 8
