# Supplementary material for: A scaffold-degradable single-rivet ASD occluder with amplatzer-like design: a multicenter randomized controlled trial
Source: Cardiovasc Interv Ther. 2026 Jun 19;41(4):1161–72. doi: 10.1007/s12928-026-01299-7 (PMC13582343; doi:10.1007/s12928-026-01299-7)
Supplement: Supplementary file 1 [file 12928_2026_1299_MOESM1_ESM.pdf]

Mallow Medical

Notes

Mallow has conducted comprehensive biological testing (including in vivo and in vitro degradation tests) on the independently developed biodegradable ventricular septal defect occluder. The materials (PDO, PET, nylon), manufacturing process, implantation site (heart) and contact mode (primarily in contact with blood, classified as long-term contact) of the biodegradable ventricular septal defect occluder used in the tests are consistent with those of the biodegradable atrial septal defect occluder submitted for approval. Therefore, differences in product specifications and dimensions do not affect the final results. The reports on the biodegradable ventricular septal defect occluder can be used to demonstrate the biological safety of the biodegradable atrial septal defect occluder.

Item	Biodegradable Atrial Septal Defect Occluder	Biodegradable Ventricular Septal Defect Occluder	Conclusion
Components	PDO degradable wire, polyester membrane and nylon suture	PDO degradable wire, polyester membrane and nylon suture	Consistent
Packaging	Primary package: blister box + coated Tyvek paper; Secondary package: aluminum foil bag; Tertiary package: colour cardboard box	Primary package: blister box + coated Tyvek paper; Secondary package: aluminum foil bag; Tertiary package: colour cardboard box	Consistent
Acceptance criteria	Test standard for incoming materials of biodegradable occluder	Test standard for incoming materials of biodegradable occluder	Consistent
Manufacturing process	Mesh weaving, connector assembly, heat treatment, membrane suturing, cleaning, primary packaging, EO sterilization, and secondary packaging, etc.	Mesh weaving, connector assembly, heat treatment, membrane suturing, cleaning, primary packaging, EO sterilization, and secondary packaging, etc.	Consistent
Contact site and duration	Atrial septal defect between the left and right atria of the heart, long-term implantation	Interventricular septum in the heart, long-term implantation	Equivalent, both implanted in the heart
Structure	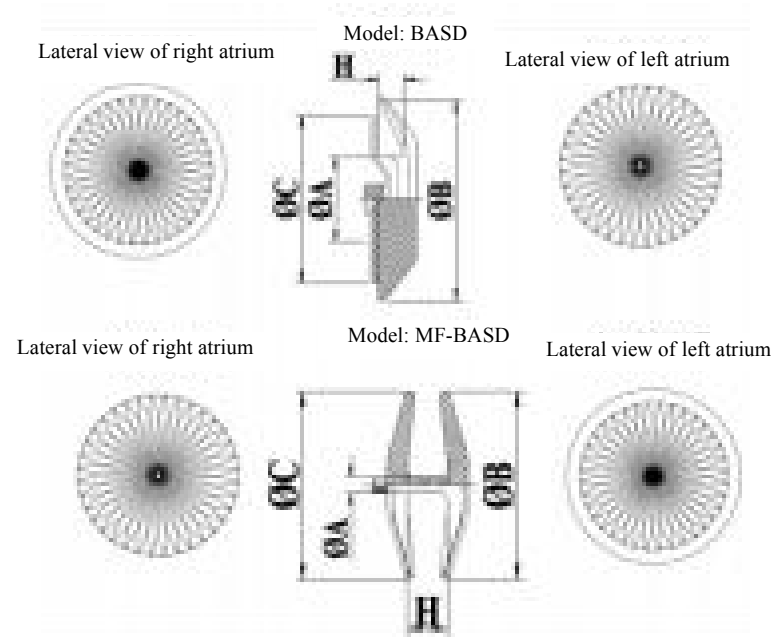	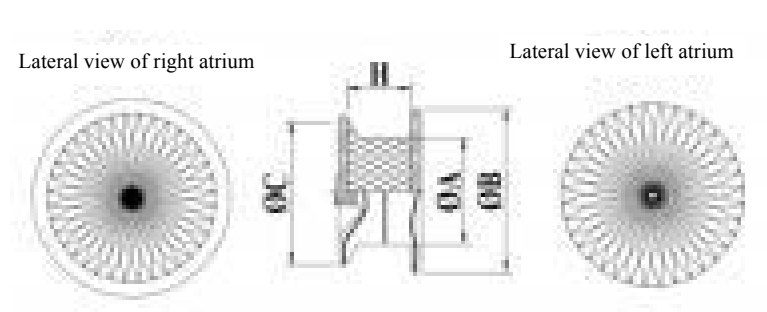	They are of single-riveted and double-disc structure, with the waist diameter designed according to the occlusion site, therefore deemed equivalent.

China National Accreditation Service
Testing
TESTING
CNAS L0001

National Institutes for Food and Drug Control

Test Report

Report No.: QH202108153

Name of Test Article:	Biodegradable Ventricular Septal Defect Occluder
Manufacturer/ Manufacturing Site:	Shanghai Mallow Medical Device Co., Ltd.
Test Purpose:	Contract testing
Reference:	GB/T16886, agreed method

Test Report from National Institutes for Food and Drug Control

Report No.: QH202108153

Name of Test Article	Biodegradable Ventricular Septal Defect Occluder	Test Article No.	QH0502201703802
Manufacturer/Manufacturing Site	Shanghai Mallow Medical Device Co., Ltd.	Lot No.	141706001
Sample Supplier	Shanghai Mallow Medical Device Co., Ltd.	Specification	/
Test Purpose	Contract testing	Dosage Form/Model	M-BVSD14-0418
Test Item	Partial test	Package Size	1 unit/bag
Date Sample Received	24 Oct 2017	Valid up to/ Expiry Date	07 Jul 2020
Quantity of Test Article	Implant: 35 sets, Finished product: 15 sets	Number of Seals	/
Reference	GB/T16886, agreed method		

Test Item	Specification	Test Result
1. Implantation and in vivo degradation testing	/	see the attachment
2. In vitro degradation testing	/	see the attachment
	Blank Below	

Remark: The contract testing is the sample testing which is carried out as per the contract on a voluntary basis by both parties. (1) Attachments: 1. Study Report on Implantation and In Vivo Degradation Testing of Biodegradable Ventricular Septal Defect Occluder; 2. Study Report on In Vitro Degradation Testing of Biodegradable Ventricular Septal Defect Occluder. (2) The testing should be completed within the shelf life of the product. (3) The in vitro degradation testing is not in the scope of our CMA and CNAS accreditation.

Testing Conclusion	This product is tested according to GB/T16886 and agreed method, and the above items are as described above.		
Authorized Signatory	[signature]	Issued Date	09 Sep 2021

Attachment 1

Study Report from National Institutes for Food and Drug Control

Implantation and In Vivo Degradation Testing (GB/T16886.6-2015)

Name of Test Article: Biodegradable Ventricular Septal Defect Occluder

Manufacturer: Shanghai Mallow Medical Device Co., Ltd.

Sponsor: Shanghai Mallow Medical Device Co., Ltd.

Study Summary

Test Sample: Degradable Ventricular Septal Defect Occluder, Lot No.: 141706001. The implantation and in vivo degradation testing was performed according to the method recommended in the Chinese national standard GB/T16886.6-2015 "Biological Evaluation of Medical Devices - Part 6: Tests for Local Effects after Implantation".

The test sample was implanted subcutaneously into animals. Similarly, the control material was implanted subcutaneously into animals. Sampling was performed at 1 week, 4 weeks, 12 weeks, 26 weeks, 39 weeks and 53 weeks after implantation for pathological observation.

Under the test conditions: (1) At 1 week, 4 weeks and 13 weeks, no irritation was observed compared to the control group. At 26 weeks, mild irritation was observed in the control group and the test group. At 39 weeks and 53 weeks, local tissue reactions were observed at the implantation sites. In the test group, significant inflammatory responses were observed, whereas there was almost no inflammatory response in the control group. Therefore, the test sample was judged to cause moderate irritation at 39 weeks and 53 weeks. However, considering that the common component, the implant wires, have been largely absorbed in both groups, while the polyester membrane fibers specific to the test group were still observed in the tissue sections, it can be inferred that the irritation in the test group may be caused by the polyester membrane fibers. (2) The degradable components in the sample were completely degraded by 26 weeks.

Conclusion

Under the test conditions: (1) At 1 week, 4 weeks and 13 weeks, no irritation was observed compared to the control group. At 26 weeks, mild irritation was observed in the control group and the test group. At 39 weeks and 53 weeks, local tissue reactions were observed at the implantation sites. In the test group, significant inflammatory responses were observed, whereas there was almost no inflammatory response in the control group. Therefore, the test sample was judged to cause moderate irritation at 39 weeks and 53 weeks. However, considering that the common component, the implant wires, have been largely absorbed in both groups, while the polyester membrane fibers specific to the test group were still observed in the tissue sections, it can be inferred that the irritation in the test group may be caused by the polyester membrane fibers. (2) The degradable components in the sample were completely degraded by 26 weeks.

Report Archiving

All raw data related to this study and a final report are archived in the Archives of National Institutes for Food and Drug Control.

Attachment 2

Study Report from National Institutes for Food and Drug Control

Study Report on In Vitro Degradation Testing

Name of Test Article: Biodegradable Ventricular Septal Defect Occluder

Manufacturer: Shanghai Mallow Medical Device Co., Ltd.

Sponsor: Shanghai Mallow Medical Device Co., Ltd.

5) Content of degradation products:

Time	Test Concentration (μg/mL)	Mean (μg/mL)	Dilution Volume (mL)	Actual Concentration (μg/mL)	Solution Volume (mL)	Amount of Degradation Products (mg)
1m-1	0.525<br>0.530	0.528	100.0	52.50	50.00	2.64
1m-2	0.431<br>0.442	0.437	100.0	43.10	50.00	2.18
1m-3	0.407<br>0.412	0.410	100.0	40.70	50.00	2.05
3m-1	4.878<br>4.887	4.883	100.0	487.80	50.00	24.41
3m-2	4.884<br>4.817	4.851	100.0	488.40	50.00	24.25
3m-3	2.779<br>2.846	2.813	100.0	277.90	50.00	14.06
6m-1	4.987<br>5.029	5.008	100.0	498.70	50.00	25.04
6m-2	6.119<br>6.160	6.140	100.0	611.90	50.00	30.70
6m-3	7.013	7.013	100.0	701.30	50.00	35.07
9m-1	17.403<br>17.453	17.428	200.0	3480.60	50.00	174.28
9m-2	14.946<br>14.657	14.802	200.0	2989.20	50.00	148.02
9m-3	15.687<br>15.9	15.806	200.0	3137.40	50.00	158.06
12m-1	24.369<br>24.502	24.436	200.0	4873.80	50.00	244.36
12m-2	24.235<br>24.415	24.325	200.0	4847.00	50.00	243.25
12m-3	24.939<br>24.8	24.886	200.0	4987.80	50.00	248.86
