# Supplementary material for: A scaffold-degradable single-rivet ASD occluder with amplatzer-like design: a multicenter randomized controlled trial
Source: Cardiovasc Interv Ther. 2026 Jun 19;41(4):1161–72. doi: 10.1007/s12928-026-01299-7 (PMC13582343; doi:10.1007/s12928-026-01299-7)
Supplement: Supplementary file 2 [file 12928_2026_1299_MOESM2_ESM.pdf]

Validation Report

Fatigue Performance of Biodegradable Atrial Septal Defect Occluder

Document No.: JK/YZ07-B04

上海形状记忆合金材料有限公司

Prepared by:	张明	Date:	2024.04.17
Reviewed by:	程海波	Date:	2024.04.17
Approved by:	黄震	Date:	2024.04.17

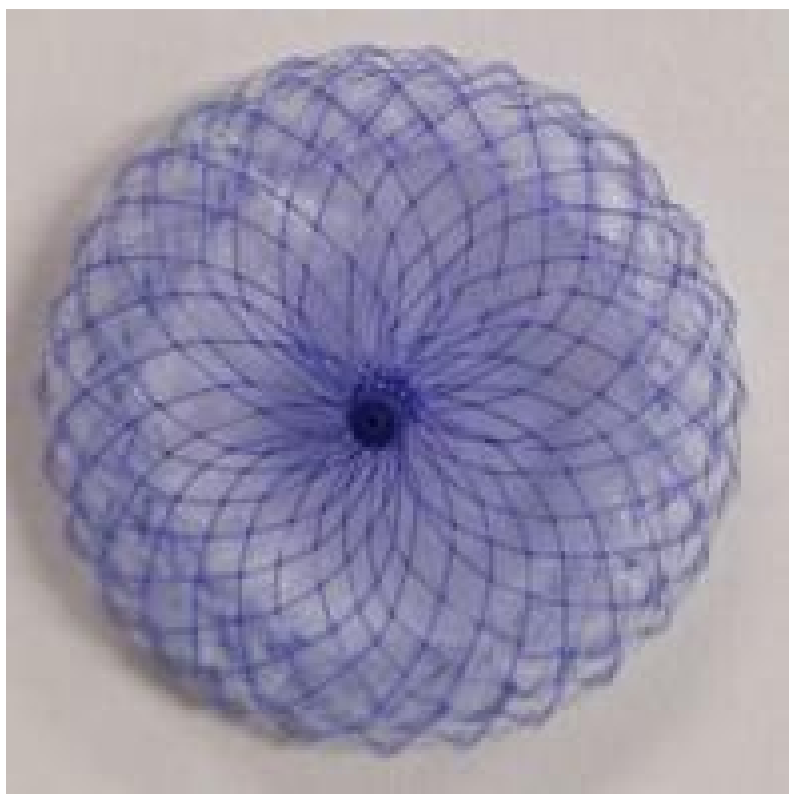

Figure 1 Image of the sample after 3.3 million cycles of testing

The sample was removed from the fatigue testing machine after 6.6 million cycles of testing (equivalent to 2 months of normal human heartbeats). As shown in the following figure, the occluder showed no signs of migration, detachment, or other abnormalities in the testing device. The mesh showed no broken wires, and there was no separation between the mesh and the connector. The interlayer membrane inside the occluder showed no rupture, maintaining its normal shape, and was securely sutured to the mesh disc, with no suture breakage observed. Furthermore, there was no significant change in the product's fixation force before and after the test.

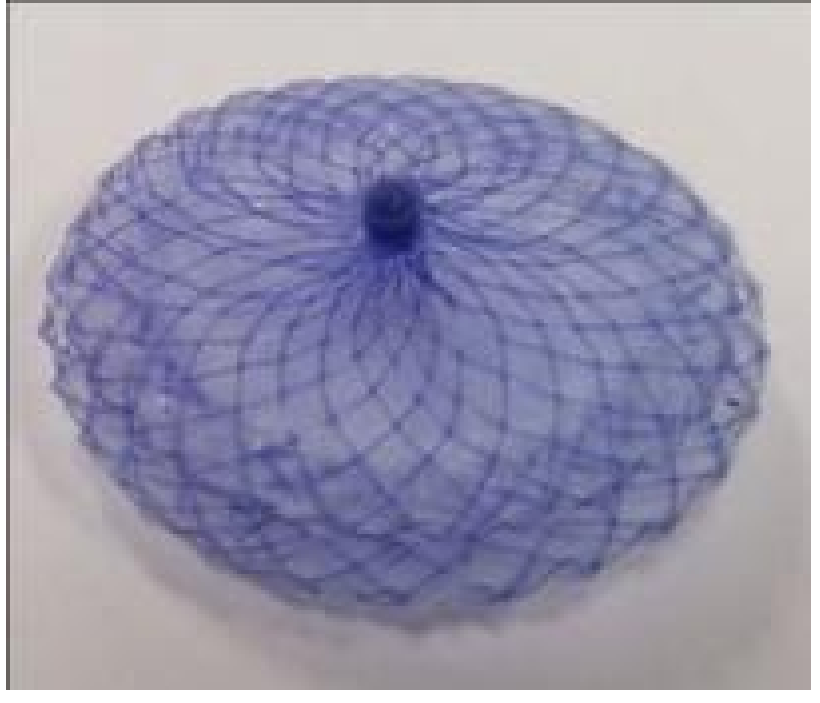

Figure 2 Image of the sample after 6.6 million cycles of testing

After 10 million cycles of fatigue testing (equivalent to 3 months of normal human heartbeats), the product showed no signs of migration, detachment, or other abnormalities in the testing device. There were only a few broken wires in the mesh, with no separation between the mesh and the connector. The interlayer membrane inside the occluder showed no rupture, maintaining its normal shape, and was securely sutured to the mesh disc, with no suture breakage observed. During the removal of the sample from the testing machine, the pulling force caused some broken wires and damage to the occluder's integrity, therefore, the subsequent fixation force testing could not be carried out.

3 Conclusion

Therefore, it can be inferred that at 1, 2, and 3 months after simulated implantation into the human body, the biodegradable atrial septal defect occluder showed no signs of migration, detachment, or other abnormalities in the testing device. The mesh showed no broken wires, and there was no separation between the mesh and the connector. The interlayer membrane inside the occluder showed no rupture, maintaining its normal shape, and was securely sutured to the mesh disc, with no suture breakage observed. Furthermore, there was no significant change in the product’s fixation force before and after the test.

Table 1:

Record of Product Life Validation Results

Sample No.	Specification	Number of Fatigue Tests Experienced	Migration or Detachment (Yes/No)	Any Broken Wires in the Mesh or between the Mesh and the Connector	Interlayer Membrane with No Rupture, Maintaining Normal Shape, Securely Sutured to the Mesh Disc with No Suture Breakage (Yes/No)	Fixation Force (Before Testing)	Fixation Force (After Testing)
1	M-BASD-04	3.3 million	No	None	Yes	1.553N	1.484N
2	M-BASD-04	6.6 million	No	None	Yes	1.708N	1.741N
3	M-BASD-04	10 million	No	1	Yes	1.654N	/
4	M-BASD-20	3.3 million	No	None	Yes	1.256N	1.190N
5	M-BASD-20	6.6 million	No	None	Yes	1.347N	1.307N
6	M-BASD-20	10 million	No	None	Yes	1.113N	/
7	M-BASD-34	3.3 million	No	None	Yes	0.917N	0.933N
8	M-BASD-34	6.6 million	No	None	Yes	0.894N	0.962N
9	M-BASD-34	10 million	No	None	Yes	0.932N	/
10	M-MF-BASD-1818	3.3 million	No	None	Yes	4.402N	4.841N
11	M-MF-BASD-1818	6.6 million	No	2	Yes	3.784N	4.245N
12	M-MF-BASD-1818	10 million	No	None	Yes	4.356N	/

Validation Report

Biodegradable Atrial Septal Defect Occluder Elastic Deformation Adaptability Testing

Document No.: JK/YZ 134-B03

Prepared by: 张明 Date: 2024.04.16

Reviewed by: [signature] Date: 2024.04.16

Approved by: [signature] Date: 2024.04.16

7.2.2.2 Results

1) Appearance: Good shape, with no obvious bulging or other abnormalities on the disc surface;

2) The dimensions of the product in each specification were within the allowable tolerance range.

3) The fixation force values are shown in the table below.

Specification	Fixation Force Setpoint (N)
M-BASD-08	0.028
M-MF-BASD-2424	0.003
M-BASD-28	0.452
M-BASD-34	0.530

4) Suture strength: After five cycles of occluder retrieval into and deployment from the sheath, there was no separation or disruption between the interlayer membrane and the mesh, and the sutures securing the mesh and interlayer membrane showed no signs of loosening or breakage. See Attachment 2 for the specific test results.

8. Conclusion

Following the above steps, the elastic adaptability testing was conducted on each model of the biodegradable atrial septal defect occluder, demonstrating that after undergoing five cycles of repeated deployment and retrieval, the occluder showed good shape in appearance, with no bulging or other abnormalities on the disc surface. Additionally, the product's dimensions, fixation force, and suture strength continued to meet the technical requirements, ensuring its safety during clinical use.
