# Supplementary material for: A scaffold-degradable single-rivet ASD occluder with amplatzer-like design: a multicenter randomized controlled trial
Source: Cardiovasc Interv Ther. 2026 Jun 19;41(4):1161–72. doi: 10.1007/s12928-026-01299-7 (PMC13582343; doi:10.1007/s12928-026-01299-7)
Supplement: Supplementary file 3 [file 12928_2026_1299_MOESM3_ESM.pdf]

上海市电子证照库
zwdtcert.sh.gov.cn

913670021100082

Filing Form for Medical Device Clinical Study

<table>
<tr><td>Study Title</td><td colspan="5">A Prospective, Multicenter, Randomized, Controlled Clinical Study on the Efficacy and Safety of Biodegradable ASD Occluder with Delivery Device</td></tr>
<tr><td>Study Objective</td><td colspan="5">To verify that the biodegradable atrial septal defect (ASD) occluder with delivery device manufactured by Shanghai Mallow Medical Device Co., Ltd is non-inferior to the conventional metallic ASD occluder with delivery device in terms of efficacy and safety in the treatment of ASD.</td></tr>
<tr><td rowspan="6">Investigational Medical Device</td><td colspan="2">Name</td><td colspan="3">Biodegradable Atrial Septal Defect Occluder with Delivery Device</td></tr>
<tr><td colspan="2">Model and Specification</td><td colspan="3">M-BASD-04, M-BASD-06, M-BASD-08, M-BASD-10, M-BASD-12, M-BASD-14 M-BASD-16, M-BASD-18, M-BASD-20, M-MF-BASD-1818, M-MF-BASD-2418, M-MF-BASD-2424, M-DS-8F-850/45, M-DS-9F-850/45, M-DS-10F-850/45, M-DS-12F-850/45, M-DS-14F-850/45, M-DS-8F-150/0, M-DS-9F-150/0, M-DS-10F-200/0, M-DS-12F-200/0, M-DS-14F-200/0</td></tr>
<tr><td rowspan="3">Category</td><td>1</td><td colspan="3">☐ Domestic Class II √ Domestic Class III
☐ Imported Class II ☐ Imported Class III</td></tr>
<tr><td>2</td><td colspan="3">☐ Active √ Passive ☐ In vitro diagnostic reagent</td></tr>
<tr><td>3</td><td colspan="3">√ Implantation ☐ Non-implantation</td></tr>
<tr><td colspan="2">Class III medical device requiring approval of clinical trial</td><td>☐ Yes
√ No</td><td>Is there any similar product in China</td><td>☐ Yes
√ No</td></tr>
</table>

Protocol Version and Date	V2.0/28 Apr 2020	Multicenter Clinical Study	√ Yes<br>☐ No
Project Start and End Dates	01 Nov 2019 - 30 Jun 2021		

Sponsor	Shanghai Mallow Medical Device Co., Ltd.	Contact	Shanghai Mallow Medical Device Co., Ltd.	Tel:	021-64019946
				Phone:	18616227703 备案专用章
Sponsor's Address	Building 15, No. 388 Yindu Road, Xuhui District, Shanghai, P.R. China			Postal Code	200231
Agent	/	Contact	/	Tel:	/
				Phone:	/
Agent's Address	/			Postal Code	/
Name of Monitor	LIU Jiaxing		Tel:	021-64019946	

Information on All Sites Involved in the Clinical Study	Information on All Investigators
1. Xinhua Hospital Affiliated to Shanghai Jiao Tong University School of Medicine; No. 1665, Kongjiang Road, Yangpu District, Shanghai; SUN Kun; 13601846338<br>(Leading site)	1. SUN Kun; Xinhua Hospital Affiliated to Shanghai Jiao Tong University School of Medicine; Pediatric Heart Center; chief physician; 13601846338

List of Materials to be Submitted	
1	Filing form for medical device clinical study
2	Letter of authorization for relevant persons authorized by the sponsor or agent to handle affairs relating to filing of the medical device clinical trial; scanned ID copies of the authorized persons
3	Scanned copy of the ethics committee approval letter
4	Scanned copy of the clinical trial agreement or contract between the sponsor and the clinical study site
5	Scanned copy of approval letter for medical device clinical trial (only for Class III medical devices requiring approval of clinical trial)

I (we) hereby declare that the contents filled in the Filing Form and the materials submitted are authentic, valid and well-documented, and are in compliance with the requirements of relevant laws and regulations. I (we) will bear corresponding legal liabilities for such contents and materials.

<table>
<tr><td colspan="2">Seal of the Sponsor:
05 Feb 2021</td></tr>
<tr><td colspan="2">Filing No.: Hu Xie Lin Bei 20210024

Special Seal for Filing of Provincial Drug Regulatory Authority
05 Feb 2021</td></tr>
<tr><td>Changes in the Start and End Dates of the Project</td><td>The end date of the project was changed to 01 Dec 2022, date of change: 27 Jul 2021</td></tr>
<tr><td>Changes in Information on Sites Involved in the Clinical Study</td><td>Added the clinical study site: Hunan Children's Hospital; No. 86 Ziyuan Road, Yuhua District, Changsha City, Hunan Province; CHEN Zhi; 13787028209; date of change: 06 May 2021
Added the clinical study site: Linyi People's Hospital; Intersection of Wohushan Road and Wuhan Road, Lanshan District, Linyi City; ZHANG Fengwei; 15969917315; date of change: 02 Jan 2022
Added the clinical study site: Beijing Children's Hospital, Capital Medical University; No. 56, Nanlishi Road, Xicheng District, Beijing; LI Zhiqiang; 13366971326; date of change: 16 Feb 2022
Added the clinical study site: The Second Xiangya Hospital of Central South University; No. 139, Renmin Middle Road, Changsha, Hunan Province; ZHAO Tianli; 18807489179; date of change: 02 Mar 2022
Added the clinical study site: The First Hospital of Jilin University; No. 71, Xinmin Street, Changchun, Jilin Province; GAO Yongsheng; 15043007511; datet of change: 07 Mar 2022
Added the clinical study site: Zhongnan Hospital of Wuhan University; No. 169 Donghu Road, Wuchang District, Wuhan City; ZHANG Gangcheng; 18963980946; date of change: 17 Jun 2022
Added the clinical study site: General Hospital of Northern Theater Command, Chinese PLA; No. 83, Wenhua Road, Shenhe District, Shenyang; WANG Qiguang; 13214255166; date of change: 13 Sep 2023</td></tr>
<tr><td>Changes in Information on Investigators</td><td>Added investigator: CHEN Zhi; Hunan Children's Hospital; Department of Cardiology; chief physician; 13787028209; date of change: 06 May 2021
Added investigator: ZHANG Fengwei; Linyi People's Hospital; Cardiac Surgery; chief physician; 15969917315; date of change: 02 Jan 2022
Added investigator: LI Zhiqiang; Beijing Children's Hospital, Capital Medical University; Cardiac Surgery; chief physician; 13366971326; date of change: 16 Feb 2022
Added investigator: ZHAO Tianli; The Second Xiangya Hospital of Central South University; Cardiac Surgery; chief physician; 18807489179; date of change: 02 Mar 2022
Added investigator: GAO Yongsheng; The First Hospital of Jilin University; Cardiac Surgery; chief physician; 15043007511; date of change: 07 Mar 2022
Added investigator: ZHANG Gangcheng; Zhongnan Hospital of Wuhan University; Department of Cardiology; chief physician; 18963980946; date of change: 17 Jun 2022
Added investigator: WANG Qiguang; General Hospital of Northern Theater Command, Chinese PLA; Department of Congenital Heart Disease; chief</td></tr>
</table>

	physician; 13214255166; date of change: 13 Sep 2023
Summary of Changes in Clinical Study Protocol	Updated the version number and date; V3.0/11 Jun 2021; date of change: 22 Jul 2021<br>Updated the version number and date; V4.0/25 Jan 2022; date of change: 24 Feb 2022<br>Updated the version number and date; V5.0/27 Mar 2023; date of change: 22 May 2023
Summary of Other Changes in the Clinical Study Filing Form	Changed the contact person to: ZHANG Jin; 18616293603; date of change: 22 Jul 2021<br>Changed the CRA to: CHAI Haihong; 13818572001; date of change: 22 Jul 2021<br>Added specifications and models of the biodegradable ASD occluder (protocol V5.0/27 Mar 2023): M-BASD-22, M-BASD-24, M-BASD-26, M-BASD-28, M-BASD-30, M-BASD-32, and M-BASD-34
Note:<br>1. The Sponsor is also the applicant for medical device registration.<br>2. A multi-center clinical study refers to a clinical study that is to be conducted at three or more clinical study sites according to the same clinical study protocol.	
