# Supplementary material for: A scaffold-degradable single-rivet ASD occluder with amplatzer-like design: a multicenter randomized controlled trial
Source: Cardiovasc Interv Ther. 2026 Jun 19;41(4):1161–72. doi: 10.1007/s12928-026-01299-7 (PMC13582343; doi:10.1007/s12928-026-01299-7)
Supplement: Supplementary file 4 [file 12928_2026_1299_MOESM4_ESM.pdf]

Approval Letter Ethics Committee of Xinhua Hospital Affiliated to Shanghai Jiaotong University School of Medicine (Amendment)

Approval No. XHEC-B-2019-006-6 **Meeting No./Date: NA**

**Protocol Title/Number**	A Prospective, Multicenter, Randomized, Controlled Clinical Study on the Efficacy and Safety of Biodegradable ASD Occluder with Delivery Device/JK-LC-BASD-01
**Reviewed Documents**	Attached Page
**Clinical Trial Approval Letter No./Clinical Trial Notification**	NA
**Study Product**	Biodegradable ASD Occluder with Delivery Device
**Phase of Study**	☐Phase I clinical trial ☐Phase II clinical trial ☐Phase III clinical trial ☐Phase IV clinical trial ■Device registration trial ☐ Post-marketing trial of medical device ☐ Registration trial of diagnostic reagent ☐ Sponsor-initiated clinical study ☐Investigator-initiated clinical study ☐Other (specify)
**Type of Review**	☐Meeting Review ■Expedited Review (Amendment • 4th) ☐Emergency Meeting Review
**Study Site /PI**	Xin Hua Hospital Affiliated to Shanghai Jiao Tong University School of Medicine Pediatric Heart Center/SUN Kun
**Sponsor/ CRO**	Shanghai Mallow Medical Device Co., Ltd.

Recommendation

Result: Approved. Please carry out this clinical trial in strict accordance with the approved study protocol and informed consent form.

Frequency of follow-up review: ☐3 Months ☐6 Months ■12 months ☐Not applicable

Ethics Committee of Xinhua Hospital Affiliated to Shanghai Jiao Tong University School of Medicine

22 May 2023

Chairman (Signature)

Notes:

1. The implementation of the approved project should comply with the protocol approved by the ethics committee (EC), as well as "Good Clinical Practice" (2020) and "Good Clinical Practice for Medical Devices" (2016) issued by National Medical Products Administration (NMPA) and National Health Commission of the People's Republic of China, Measures for Ethical Review of Life Science and Medical Research Involving Humans" (2023) issued by the National Health Commission, WMA "Declaration of Helsinki" (2013), CIOMS/WHO "International Ethical Guidelines for Health-Related Research Involving Humans" (2016), ICH-GCP and other relevant laws and regulations.
2. This clinical trial should be implemented within one (1) year since the date of approval by the EC, and this approval letter becomes invalid spontaneously after the expiration date.
3. Any modification to the study protocol, the informed consent form and other relevant documents during the study must obtain an approval from the Ethics Committee before implementation.
4. To eliminate emergency hazards to the subjects, modifications to or deviations from the study protocol by the investigator without the approval of the EC should be reported to the EC and the Sponsor timely and justified, and should be reported to drug regulatory authorities if necessary.
5. The investigator should read the Sponsor-provided safety information relating to the clinical trial promptly upon receipt, and consider the necessity of making corresponding adjustments to the subject's treatment. When necessary, the investigator should communicate with the subject as soon as possible, and report the Sponsor-provided suspected and unexpected serious adverse reactions (SUSARs) to the EC.
6. If the clinical trial is terminated or suspended by the Sponsor, the investigator should report it to the clinical study site and the EC immediately and provide detailed explanations in writing.
7. If the approved clinical trial is terminated or suspended by the EC, the investigator should report it to the clinical study site and the Sponsor immediately and provide detailed explanations in writing.
8. The investigator should submit an annual report of the clinical study to the EC.
9. In case of any changes that may significantly affect the conduct of the clinical trial or result in an increased risk to the subjects, the investigator should submit a written report to the Sponsor, the EC and the clinical study site promptly.
10. Upon completion of the clinical study, the investigator should provide the EC with a summary of the clinical study results.

Address: No. 1665 Kongjiang Road Shanghai, P.R China.Postcode:200092 Tel: +86-21-25076143
Fax: +86-21-25078922

Attached Page

A Prospective, Multicenter, Randomized, Controlled Clinical Study on the Efficacy and Safety of Biodegradable ASD Occluder with Delivery Device

List of reviewed documents

S/N	Document Name	Version	Date
1	List of Application Dossiers for the Amendment	/	/
2	Application Form for Amendment Review	/	/
3	Comparison Table of Amendments to the Clinical Study Protocol	/	/
4	Comparison Table of Amendments to the Case Report Form	/	/
5	Comparison Table of Amendments to Study Medical Records	/	/
6	Comparison Table of Amendments to the Investigator's Brochure	/	/
7	Comparison Table of Amendments to the Package Insert of Biodegradable ASD Occluder	/	/
8	Clinical Study Protocol	V5.0	27 Mar 2023
9	Case Report Form	V5.0	27 Mar 2023
10	Study Medical Records	V4.0	27 Mar 2023
11	Investigator's Brochure	V4.0	27 Mar 2023
12	Package Insert of Biodegradable ASD Occluder	Version 3	27 Mar 2023
13	Investigator's Brochure	V1.0	28 Apr 2020
14	Package Insert of ASD Occluder Delivery Device (45 °)	Version 2	09 Jan 2019
15	Registration Certificate of ASD Occluder Delivery Device (45 °)	NMPA Medical Device Approval No.	20193030415
16	Package Insert of ASD Occluder Delivery Device (0°)	Version 3	30 Aug 2022
17	Registration Certificate of ASD Occluder Delivery Device (0°)	NMPA Medical Device Approval No.	20223030793
18	Technical Requirements for ASD Occluder Delivery Device (0°)	/	/
19	Package Insert of the Control Device Used in the Clinical Trial	Version 4	18 Nov 2019
20	Registration Certificate of Control Device Used in the Clinical Trial	NMPA Medical Device Approval No.	20153130031
21	Test Report for Biodegradable Atrial Septal Occluder (new specification)	NMPA Medical Test (Device)	QW2022 No. 6822
22	Technical Requirements for Biodegradable Atrial Septal Occluder (new specification)	/	
23	Notes to Change of the Statistical Analysis Facility for the Clinical Project		

Ethics Committee of Xinhua Hospital Affiliated to Shanghai Jiao Tong University School of Medicine
22 May 2023

Address: No. 1665 Kongjiang Road Shanghai, P.R China.Postcode:200092 Tel: +86-21-25076143
Fax: +86-21-25078922
