# Supplementary material for: A scaffold-degradable single-rivet ASD occluder with amplatzer-like design: a multicenter randomized controlled trial
Source: Cardiovasc Interv Ther. 2026 Jun 19;41(4):1161–72. doi: 10.1007/s12928-026-01299-7 (PMC13582343; doi:10.1007/s12928-026-01299-7)
Supplement: Supplementary file 5 [file 12928_2026_1299_MOESM5_ESM.pdf]

Informed Consent Form for A Prospective, Multicenter, Randomized, Controlled Clinical Study on the Efficacy and Safety of Biodegradable ASD Occluder with Delivery Device

(Legal Guardian Version)

Name of Investigational Medical Device: Biodegradable Atrial Septal Defect Occluder with Delivery Device

Protocol Title: A Prospective, Multicenter, Randomized, Controlled Clinical Study on the Efficacy and Safety of Biodegradable ASD Occluder with Delivery Device

Sponsor: Shanghai Mallow Medical Device Co., Ltd.

Dear Parent or Legal Guardian,

Your child will be invited to participate in a study: a prospective, multicenter, randomized, controlled clinical study on the efficacy and safety of biodegradable ASD occluder with delivery device. Your child's participation in this study is entirely voluntary. This informed consent form will provide you with important information about the study and you are advised to read it carefully before making a decision if you want your child to take part in this study or not. If you have any questions or if there is anything not clearly understood, please ask the study doctor responsible for the study project, and the study team will answer any questions you have raised. If you consent to your child's participation in this clinical study, please sign the statement in this document.

1. Study Background and Objective

(1) Study Background

Currently, the atrial septal defect occluders marketed in China are all made of metal materials and once implanted, they will remain in the body permanently. In spite of good therapeutic effects, they are also associated with some complications, such as occluder detachment, thrombosis and allergy to nickle among others.

The control device in this study is the marketed "Single-riveted Atrial Septal Defect Occluder with Delivery Device" manufactured by Shanghai Mallow Medical Device Co., Ltd.

The investigational device is "Biodegradable Atrial Septal Defect Occluder with Delivery Device (not marketed yet)". The biodegradable ASD occluder is knitted by degradable wire. After implantation into the human body, the autologous cell tissue at the defect site grows and is endothelialized. The supporting structure of the occluder can be gradually absorbed by the body and degraded into carbon dioxide and water which are harmless to the body. In this way, the defect is completely repaired by the own tissue of the body. The experimental results in animals showed that the neointimal tissue gradually covered the device from the edge at 30 days after operation; the device was almost completely covered by neointimal tissue at 90 days after operation; and the occluder was completely covered and fused by smooth and dense intimal tissue at 180 days after operation.

This is a prospective, multicenter, randomized, controlled clinical study, in which the non-marketed biodegradable ASD occluder with delivery device manufactured by Shanghai Mallow Medical Device Co., Ltd will be investigated against the marketed metallic ASD occluder with delivery device manufactured by Shanghai Mallow Medical Device Co., Ltd to compare their efficacy and safety.

(2) Study Objective

To verify that the non-marketed biodegradable atrial septal defect occluder with delivery device manufactured by Shanghai Mallow Medical Device Co., Ltd is not inferior to the marketed single-riveted metallic atrial septal defect occluder with delivery device manufactured by Shanghai Mallow Medical Device Co., Ltd in terms of efficacy and safety after implantation into the human body.

2. Study Methodology and Contents

(1) Study Methodology

The biodegradable ASD occluder or metallic single-riveted ASD occluder is delivered through the delivery device to ASD for occlusion by minimally invasive interventional technique under the guidance of ultrasound or DSA imaging.

(2) Study Contents

① Signing of informed consent form:

If you and your child decide to take part in this study, we will ask you to sign this informed consent form before we carry out any study-related activities. If a new version of this document is generated during the course of the study, you will be asked to sign it again.

② Pre-operative examination:

The study will begin with screening examination after you have voluntarily signed the informed consent form approved by the ethics committee (EC). The purpose of the screening examination is to find out if your child meets the requirements for taking part in the study.

During the screening examination, the following will be done: collection of medical history and demographic data, physical examination, hematology, liver function test, renal function test, coagulation function test, pregnancy test (not required for participants before the age of menarche), urinalysis, ultrasonography and ECG examination. The doctor will draw about 5 - 6 mL of blood from your child. Urine samples (a small cup of urine) will be also collected from your child for urine test (urinalysis).

③ Case screening:

Following the completion of the screening examination, the study doctor will determine your child's eligibility to continue in the study based on the inclusion and exclusion criteria outlined

below. If your child is not eligible to continue in the study, the study doctor will give you explanations and discuss other treatment options with you.

The test reports of your child obtained within 15 days before the signing of the consent document are also acceptable as screening results.

Inclusion Criteria:

Internal medicine: (To be eligible, the subject has to meet either criterion #2 or #3 and all of the remaining criteria)

1. Age ≥ 2 years.
2. An atrial septal defect (ASD) in diameter ≥ 5 mm and ≤ 36 mm, carrying a left-to-right shunt, with the evidence of increased right-heart volume load.
3. Small defect, with a transient right-to-left shunt at the atrial level and suspected embolic sequelae (stroke or recurrent transient ischemic attack).
4. The distance ≥ 5 mm from the edge of an ASD to the coronary sinus, the superior and inferior vena cavae and the the pulmonary vein, and ≥ 7 mm to the atrioventricular valve.
5. A diameter of the atrial septum greater than that on the left atrium side of the selected occluder.
6. The subject is voluntary to take part in the clinical trial, signs the Informed Consent Form (ICF) and agrees to complete the follow-up procedures as required.

Surgery: (To be eligible, the subject has to meet either criterion #2 or #3 and all of criteria #1, #4, #5 and #8, but not necessarily all of the remaining)

1. Age ≥ 6 months and weight ≥ 6 kg;
2. Ostium secundum ASD ≥ 5 mm and ≤ 36 mm in diameter, carrying a left-to-right shunt, with the evidence of increased right-heart volume load.
3. Small defect, with a transient right-to-left shunt at the atrial level and suspected embolic sequelae (stroke or recurrent transient ischemic attack).
4. The distance ≥ 5 mm from the edge of an ASD to the coronary sinus, the superior and inferior vena cavae and the the pulmonary vein, and ≥ 7 mm to the atrioventricular valve.
5. A diameter of the atrial septum greater than that on the left atrium side of the selected occluder.
6. Other cardiac malformations, if present, do not reject treatment by extracorporeal circulation.
7. Post-operative residual shunts.
8. The subject is voluntary to take part in the clinical trial, signs the Informed Consent Form (ICF) and agrees to complete the follow-up procedures as required.

Exclusion Criteria: (The subjects will be excluded from the study if they meet any of the criteria)

Internal medicine:

- Right-to-left shunts due to severe pulmonary arterial hypertension.

- Patients with other cardiovascular malformations who are expected to undergo a surgical procedure within one year.
- Presence of sepsis within the last one month, or presence of a systemic infection that has not been cured.
- Patients with haemorrhagic disorder, ulcer, or other conditions that do not allow maintenance treatment with aspirin and other antiplatelet agents for the coming 6 months.
- Patients with known hypercoagulability.
- Patients with intracardiac thrombus (especially in the left atrium or left atrial appendage) as shown by echocardiography, and venous thrombosis at the insertion site of catheter.
- Patients with other infectious diseases that make cardiac catheterization unsuitable.
- Patients with a distance < 5 mm from the edge of an ASD to the coronary sinus, the atrioventricular valve, the right superior pulmonary vein and the superior vena cava.
- Pregnant or breast-feeding subjects, or subjects planning to become pregnant who do not agree to use effective methods of contraception during the trial.
- Patients who have participated in other clinical trials that would affect evaluation of the investigational medical device and are remaining in or not withdrawn from such trials by the first 3 months of the screening period in the present study;
- Patients with life expectancy of less than 1 year due to high-risk diseases such as tumor.
- Patients who, in the opinion of the investigator, are not suitable for this clinical study.
- Patients who refuse to give their informed consent.

Surgery:

- Ostium primum ASD and sinus venosus ASD.
- Endocarditis and haemorrhagic disorder.
- Thrombus at the placement site of occluder, and venous thrombosis at the insertion site of catheter.
- Right-to-left shunts due to severe pulmonary arterial hypertension.
- Severe myocardial or valvular disease unrelated to ASD.
- History of infectious diseases within the past one month, or presence of uncontrolled infectious diseases.
- Patients with haemorrhagic disorder, or active gastric/duodenal ulcers that are not cured.
- Thrombus in the left atrium or left atrial appendage, partial or total anomalous pulmonary venous drainage, and left intra-atrial septum, left atrial or left ventricular dysplasia.
- Pregnant or breast-feeding subjects, or subjects planning to become pregnant who do not agree to use effective methods of contraception during the trial.

- Patients who have participated in other clinical trials that would affect evaluation of the investigational medical device and are remaining in or not withdrawn from such trials by the first 3 months of the screening period in the present study;
- Patients with life expectancy of less than 1 year due to high-risk diseases such as tumor.
- Patients with other cardiovascular malformations who are expected to undergo a surgical procedure within one year.
- Patients who, in the opinion of the investigator, are not suitable for this clinical study.
- Patients who refuse to give their informed consent.

④ Randomization:

The study will be conducted at designated clinical sites and 112 patients will be enrolled in the study. The study is a randomized controlled trial. If you meet the inclusion criteria, the study doctor will assign you a randomization number in random order, open the corresponding randomization envelope and then assign you to the test group or control group according to the envelope.

Test group: Biodegradable ASD occluder with delivery device (not marketed yet);

Control group: Single-riveted ASD occluder with delivery device (marketed)

The single-riveted ASD occluder manufactured by Shanghai Mallow Medical Device Co., Ltd is made of nickel-titanium alloy wire and has a single-riveted structure with built-in interlayer membrane. This occluder is designed to achieve occlusion of the defect through mechanical blocking of the blood flow by placing the nickle-titanium mesh structure with built-in interlayer membrane at the lesion. The control occluder has been proved in clinical trials and has been marketed, with a good efficacy and safety profile in clinical setting.

⑤ Implementation of ASD closure:

The study doctor will select the invetigational or control device to perform ASD closure for you depending on your randomization result and record the associated complications and device use.

⑥ Post-operative follow-up:

In this study, you will need to undergo the following examinations from post-operation to discharge, and at 4 visits to the hospital after surgery.

- Post-operation to discharge: ultrasonography and electrocardiogram (ECG) examination.
- 1 month ± 7 days after operation: ultrasonography, ECG examination, hematology, urinalysis, and blood pregnancy test (not required for participants before the age of menarche or males).
- 3 months ± 30 days after operation: ultrasonography, ECG examination, urinalysis, and blood pregnancy test (not required for participants before the age of menarche or males).
- 6 months ± 30 days after operation: ultrasonography, ECG examination, urinalysis, and blood pregnancy test (not required for participants before the age of menarche or males)

- 12 months ± 30 days after operation: ultrasonography, ECG examination, hematology, urinalysis, liver function test, renal function test, and blood pregnancy test (not required for participants before the age of menarche or males).

About 3 - 6 mL of blood will be collected from you for hematology, and urine samples (a small cup of urine) will also be collected for urinalysis.

3. Study Process and Duration

Your participation in this study is expected to last approximately 13 months, requring you to return to the hospital for follow-up examinations at the following time points: from post-operation to discharge, 1 month ± 7 days after operation, 3 months ± 30 days after operation, 6 months ± 30 days after operation, and 12 months ± 30 days after operation.

4. Potential Benefits

(1) Treatment of your congenital heart disease of atrial septal defect may improve your condition or may not benefit you.

(2) You doctor will give you professional medical care and perform close follow-up to guide your recovery.

(3) Your participation may provide a scientific basis for the approval of the investigational device for marketing, and may help patients with the same disease as you.

5. Possible Risks and Discomforts

Risks associated with collection of biological samples: during the study, your blood and urine samples will be collected. The collection procedures will be performed by relevant nurses or specialists in strict accordance with the standard operating procedure (SOP). However, there may still be physiological discomfort such as pain, local cyanosis, bleeding and local infection, as well as psychological discomfort such as pain feeling, psychological fear and loneliness away from your family.

Possible risks associated with medical history collection: there may be some psychological discomfort for you to have communication and talks with us.

Both the investigational and control devices may cause the same surgical risks, including complications such as residual shunts, thromboembolism, gas embolism, headache or migraine, puncture site haematoma and femoral arteriovenous fistula, cardiac tamponade, occluder migration or detachment, arrhythmia, aorta-to-right-atrium-and-left-atrium fistula, and hemolysis, as well as the risk of death. In case of any complications, the study doctor will provide effective treatment based on relevant risks and actions to be taken as described in "Chinese Expert Consensus on Interventional Treatment of Common Congenital Heart Diseases - Interventional Treatment of Atrial Septal Defect", "2015 Expert Consensus on Interventional Treatment of Common Congenital Heart Diseases in Children", "Expert Consensus on Interventional Management of Common Cardiovascular Diseases by Surgical Approach", and "Guidelines for Transcatheter Interventions on Congenital Heart Diseases". We guarantee that we will take more effective and reasonable risk prevention and management measures to minimize the surgical risks.

In addition, since the investigational device is made of degradable materials, if you are assigned to the test group, you may also have the following risks: inflammatory reactions due to degradation of the device, and residual shunts due to possible incomplete cover of the defect by neointimal tissue cells.

Reproductive risk: You need to use effective contraceptions during the study. If you have a positive pregnancy test, you are not allowed to take part in this study.

6. Treatment and Financial Compensation for Study-related Injuries

If you experience an injury during your participation in the study or an adverse event during the treatment period, please contact your study doctor, and you will be given treatment in a timely manner. The sponsor, Shanghai Mallow Medical Device Co., Ltd., has bought a clinical medical insurance for this study. In case of any study-related injury or death during the course of the study, corresponding treatment expenses and compensation will be covered by the Sponsor or paid by the insurance company according to relevant laws.

Even if you have signed this informed consent form, you still retain all of your legal rights.

7. Alternative Treatments Other Than the Current Study

If you do not participate in this clinical trial, you may choose to receive treatment with other marketed medical devices that have been approved by the National Medical Products Administration (NMPA), e.g., marketed metallic ASD occluders.

In addition, you may also choose to undergo a surgical procedure for repair.

8. Confidentiality of Medical Records

We will make every effort to protect your privacy as far as the law is concerned. Any public report concerning this study will not mention your personal information Case data from this study may be reviewed by the investigator, the ethics committee (EC), Shanghai Mallow Medical Device Co., Ltd., government departments, or the institution(s) overseeing the conduct of this study.

All your study-related documents will be distinguished by a code. In this way, you will not be identified in any way in all reports and papers about this study. All data that establish links to your privacy will be kept in a secured place at the site (department) of the investigator, i.e., the hospital where the study is conducted. The study data will be retained for at least 10 years.

9. Treatments and Other Relevant Subsidies That May Become Available During the Study

When you participate in this study, the "biodegradable atrial septal defect occluder with delivery device" or "atrial septal defect occluder with delivery device" used during the surgical procedure is provided for free. Examinations/tests required during the surgical procedure and post-operation follow-up as specified in the study protocol, such as hematology, urinalysis, liver function test, renal function test, coagualation function test, pregnancy test (not required for participants before the age of menarche), ECG and Doppler ultrasound examination, are also provided for free. In addition, during the follow-up period (4 visits), you will be paid 200 yuan per follow-up visit for the travel expenses incurred due to travel to the medical facility for examination/tests.

However, you will not be subsidized for taking part in this study.

10. Voluntary Participation in and Withdrawal from the Study

Participation in this study is entirely voluntary. You may refuse to participate in the study, and you are free to withdraw from the study at any time during the course of the study. Withdrawal from the study will not result in a penalty, discrimination or retaliation, and will not adversely affect your future medical care and rights.

If you want to withdraw from the study, you should tell us. In this case, we will arrange end-of-study examinations/tests for you to ensure that you will end the study in the safest way.

The study doctor or the sponsor may decide to withdraw you from the study without your consent, if:

- You fail to follow the requirements of the study protocol and the instructions from the study team members of the project;
- The study doctor believes that the study will not provide you with the greatest benefit;
- The study is stopped by the sponsor, the ethics committee (an organization that reviews the study to protect your rights and interests), or a regulatory agency.

11. Requirements for Participation in the Study

When participating in this clinical study, you need to follow the instructions of the study staff throughout the clinical study and should:

- Cooperate with the study doctor as much as possible to complete each visit and relevant examinations/tests on time;
- Seek the study doctor's advice in advance or inform the study doctor timely later if you need to receive any other treatment due to a change of your condition;
- Follow the medical advice and do not use any other prohibited drug/device during the course of the study, and should inform the study doctor timely if you have to use such a drug/device.

12. What If New Information Emerges

During the study, you will be contacted in a timely manner if there is any significant new development or new medical information relating to your health, for example, advising you to take some further examinations/tests to confirm such new information.

Based on such new information, your study doctor may also advise you to withdraw from the study. The study doctor will explain the reason(s) to you and discuss with you how to better treat your disease.

13. Contact Information

You can ask questions at any time about anything that is not clearly understood by you, and the project team will answer all your questions raised before, during, and after the study. If you feel that your questions are not fully answered, or if the answers are not clearly understood by you, you may ask questions again until they are answered to your satisfaction. The contact information is as follows:

Doctor's Name: ____________________ Contact Number: __________________

If you have questions about your rights as a study participant, or if you want to get or provide information, or if you want to have a talk with someone who is not directly involved in the study, you may contact

the ethics committee at (phone number): ________________

Informed Consent Form Signature Page

Statement of Subject's Legal Guardian:

I have read and understand the information in this informed consent form. I have had an opportunity to ask questions and I am satisfied with the answers to all the questions. I have been given enough time and opportunity to ask questions about the details of the study and to consider whether to take part in the study or not. I voluntarily consent that my child will participate in this study. I do not give up any of my legal rights by signing this consent document.

I have been informed that I will receive a signed copy of this document.

Name of Child (Print):________________________________

Signature of Parent/Legal Guardian (Print): _______________Date: DD MMM YYYY

Contact Number:_____________________________________

Statement of Investigator:

I confirm that I have explained to the subject's legal guardian the details of this study, including his/her rights, as well as possible benefits and risks, and have given him/her a copy of the signed informed consent document.

Signature of Investigator (Print): ________________________Date: DD MMM YYYY

Contact Number:________________________________
