# Supplementary material for: A scaffold-degradable single-rivet ASD occluder with amplatzer-like design: a multicenter randomized controlled trial
Source: Cardiovasc Interv Ther. 2026 Jun 19;41(4):1161–72. doi: 10.1007/s12928-026-01299-7 (PMC13582343; doi:10.1007/s12928-026-01299-7)
Supplement: Supplementary file 6 [file 12928_2026_1299_MOESM6_ESM.pdf]

Protocol for A Prospective, Multicenter, Randomized, Controlled Clinical Study on the Efficacy and Safety of Biodegradable ASD Occluder with Delivery Device

Name of Investigational Medical Device: Biodegradable Atrial Septal Defect Occluder with Delivery Device

Model/Specification: See the Protocol for details

Management Classification of Investigational Medical Device: Class III

Class III medical device requiring approval of clinical trial Yes□ No ■

Is there any similar product in China Yes□ No ■

Protocol No.: JK-LC-BASD-01

Protocol Version No. and Date: V5.0, dated 27 Mar 2023

Leading Clinical Trial Site: Xin Hua Hospital Affiliated to Shanghai Jiao Tong University School of Medicine

Coordinating Investigator: SUN Kun

Statistical Analysis Facility: Institute of Basic Medical Sciences, Chinese Academy of Medical Sciences

Sponsor: Shanghai Mallow Medical Device Co., Ltd.

Confidentiality Statement

All information contained herein is confidential and shall be kept confidential by any personnel who have access to such information. Without the written permission of the sponsor, such information may not be used for the conduct of any other study, or for development or any other commercial purposes. Where the information contained herein must be used in a scientific article for publication, prior written consent of the sponsor must be obtained. If a violation of this confidentiality statement and a situation involving authority not granted by China's laws and/or regulations, it will be settled by means of arbitration or appeal to the court where the Sponsor is located.

Protocol Synopsis

Study Title	A Prospective, Multicenter, Randomized, Controlled Clinical Study on the Efficacy and Safety of Biodegradable ASD Occluder with Delivery Device
Sponsor	Shanghai Mallow Medical Device Co., Ltd.
Device Name	Biodegradable Atrial Septal Defect Occluder with Delivery Device
Device Classification	Class III medical device
Study Objective	To verify that the biodegradable atrial septal defect (ASD) occluder with delivery device manufactured by Shanghai Mallow Medical Device Co., Ltd is non-inferior to conventional metal ASD occluders and their delivery devices in terms of efficacy and safety in the treatment of ASD.
Study Design	This study is designed as a prospective, multicenter, randomized, controlled non-inferiority trial
Sample Size	In this study, the active-controlled non-inferiority design was used. By referring to references [7-11] and the results of the clinical trial for registration of the nickel-titanium ASD device of Mallow, the investigator concluded that the success rate of closure would be up to 98% to 100% at 6 months after operation. Taking α = 0.025 (one-sided), β = 0.2 (power of test: 80%), a non-inferiority margin δ = -10% and a randomization ratio of 1:1 between the test group and the control group, the sample size is calculated using the conventional large-sample normal approximation formula:<br>$n_t = n_c = \frac{(z_{1-\alpha} + z_{1-\beta})^2 (p_t(1-p_t) + p_c(1-p_c))}{[p_t - p_c - \delta)]^2}$<br>Similarly, calculated using the professional software PASS14 for calculation of sample size, 32 eligible subjects are required per group.<br>Considering the feasibility and the special statistical method and corresponding adjustment (35 to 45 cases are required) when the rate is high (e.g., 100%), the number of eligible subjects in each group is determined to be 45 in this study. Moreover, considering the drop-out rate of subjects during the trial (about 20% in this study), a total of 112 subjects are planned to be enrolled, that is, 56 subjects in the test group and the control group each.
Study Period	An application for registration will be submitted to National Medical Products Administration (NMPA) after 6 months ± 30 days of observation on the efficacy endpoint. After the end of study, follow-up observation will continue for the subjects until 12 months ± 30 days post-operation.
Inclusion Criteria	Internal medicine: (To be eligible, the subject has to meet either criterion #2 or #3 and all of the remaining criteria)<br>1. Age ≥ 2 years.<br>2. An atrial septal defect (ASD) in diameter ≥ 5 mm and ≤ 36 mm, carrying a left-to-right shunt, with the evidence of increased right-heart volume load.<br>3. Small defect, with a transient right-to-left shunt at the atrial level and suspected embolic sequelae (stroke or recurrent transient ischemic attack).<br>4. The distance ≥ 5 mm from the edge of an ASD to the coronary sinus, the superior and inferior vena cavae and the pulmonary vein, and ≥ 7 mm to the atrioventricular valve.<br>5. A diameter of the atrial septum greater than that on the left atrium side of the selected occluder.<br>6. The subject is voluntary to take part in the clinical trial, signs the Informed Consent Form (ICF) and agrees to complete the follow-up procedures as required.<br>Surgery: (To be eligible, the subject has to meet either criterion #2 or #3 and all of criteria #1, #4, #5 and #8, but not necessarily all of the remaining)<br>1. Age ≥ 6 months and weight ≥ 6 kg;<br>2. Ostium secundum ASD ≥ 5 mm and ≤ 36 mm in diameter, carrying a left-to-right shunt, with the evidence of increased right-heart volume load.<br>3. Small defect, with a transient right-to-left shunt at the atrial level and suspected embolic sequelae (stroke or recurrent transient ischemic attack).<br>4. The distance ≥ 5 mm from the edge of an ASD to the coronary sinus, the superior

	and inferior vena cavae and the pulmonary vein, and ≥ 7 mm to the atrioventricular valve.<br>5. A diameter of the atrial septum greater than that on the left atrium side of the selected occluder.<br>6. Other cardiac malformations, if present, do not reject treatment by extracorporeal circulation.<br>7. Post-operative residual shunts.<br>8. The subject is voluntary to take part in the clinical trial, signs the Informed Consent Form (ICF) and agrees to complete the follow-up procedures as required.
Exclusion Criteria (The subjects will be excluded from the study if they meet any of the criteria)	Internal medicine:<br>1. Right-to-left shunts due to severe pulmonary arterial hypertension.<br>2. Patients with other cardiovascular malformations who are expected to undergo a surgical procedure within one year.<br>3. Presence of sepsis within the last one month, or presence of a systemic infection that has not been cured.<br>4. Patients with haemorrhagic disorder, ulcer, or other conditions that do not allow maintenance treatment with aspirin and other antiplatelet agents for the coming 6 months.<br>5. Patients with known hypercoagulability.<br>6. Patients with intracardiac thrombus (especially in the left atrium or left atrial appendage) as shown by echocardiography, and venous thrombosis at the insertion site of catheter.<br>7. Patients with other infectious diseases that make cardiac catheterization unsuitable.<br>8. Patients with a distance < 5 mm from the edge of an ASD to the coronary sinus, the atrioventricular valve, the right superior pulmonary vein and the superior vena cava.<br>9. Pregnant or breast-feeding subjects, or subjects planning to become pregnant who do not agree to use effective methods of contraception during the trial.<br>10. Patients who have participated in other clinical trials that would affect evaluation of the investigational medical device and are remaining in or not withdrawn from such trials by the first 3 months of the screening period in the present study.<br>11. Patients with life expectancy of less than 1 year due to high-risk diseases such as tumor.<br>12. Patients who, in the opinion of the investigator, are not suitable for this clinical study.<br>13. Patients who refuse to give their informed consent.<br>Surgery:<br>1. Ostium primum ASD and sinus venosus ASD.<br>2. Endocarditis and haemorrhagic disorder.<br>3. Thrombus at the placement site of occluder, and venous thrombosis at the insertion site of catheter.<br>4. Right-to-left shunts due to severe pulmonary arterial hypertension.<br>5. Severe myocardial or valvular disease unrelated to ASD.<br>6. History of infectious diseases within the past one month, or presence of uncontrolled infectious diseases.<br>7. Patients with haemorrhagic disorder, or active gastric/duodenal ulcers that are not cured.<br>8. Thrombus in the left atrium or left atrial appendage, partial or total anomalous pulmonary venous drainage, and left intra-atrial septum, left atrial or left ventricular dysplasia.<br>9. Pregnant or breast-feeding subjects, or subjects planning to become pregnant who do not agree to use effective methods of contraception during the trial.<br>10. Patients who have participated in other clinical trials that would affect evaluation of the investigational medical device and are remaining in or not withdrawn from such trials by the first 3 months of the screening period in the present study.<br>11. Patients with life expectancy of less than 1 year due to high-risk diseases such as tumor.<br>12. Patients with other cardiovascular malformations who are expected to undergo a

	surgical procedure within one year.<br>13. Patients who, in the opinion of the investigator, are not suitable for this clinical study.<br>14. Patients who refuse to give their informed consent.
Investigational Device	Test group<br>Name: Biodegradable Atrial Septal Defect Occluder<br>Specification and Model: M-BASD-04, M-BASD-06, M-BASD-08, M-BASD-10, M-BASD-12, M-BASD-14, M-BASD-16, M-BASD-18, M-BASD-20, M-BASD-22, M-BASD-24, M-BASD-26, M-BASD-28, M-BASD-30, M-BASD-32, M-BASD-34, M-MF-BASD-1818, M-MF-BASD-2418, and M-MF-BASD-2424<br>Control group<br>Name: Single-riveted Atrial Septal Defect Occluder<br>Specification and Model: M-ASD-04, M-ASD-06, M-ASD-08, M-ASD-10, M-ASD-12, M-ASD-14, M-ASD-16, M-ASD-18, M-ASD-20, M-ASD-22, M-ASD-24, M-ASD-26, M-ASD-28, M-ASD-30, M-ASD-32, M-ASD-34, M-ASD-36, and M-ASD-38<br>Name: Atrial Septal Defect Occluder Delivery Device<br>Specification and Model: M-DS-6F-850/45, M-DS-7F-850/45, M-DS-8F-850/45, M-DS-9F-850/45, M-DS-10F-850/45, M-DS-12F-850/45, M-DS-14F-850/45, M-DS-6F-150/0, M-DS-7F-150/0, M-DS-8F-150/0, M-DS-9F-150/0, M-DS-10F-200/0, M-DS-12F-200/0, and M-DS-14F-200/0
Description of Safety Parameters	Complications: defined as any abnormal reactions that may occur during or after the surgical procedure. Mainly include:<br>①. Moderate or greater residual shunts<br>②. Thromboembolism<br>③. Gas embolism<br>④. Persistent or prolonged headache/migraine<br>⑤. Puncture site haematoma and femoral arteriovenous fistula<br>⑥. Tamponade cardiac<br>⑦. Occluder migration or detachment<br>⑧. Arrhythmia associated with the occluder<br>⑨. Aorta-to-right- and left-atrium fistula<br>⑩. Hemolysis<br>⑪. Other less common complications
Description of Efficacy Parameters	①. Device success rate<br>"Device success" is defined as follows: the occluder is successfully delivered and implanted at the intended target site, with echocardiography confirming correct position of the implanted device. The delivery device operates smoothly, is easy to handle, and allows accurate positioning, with the device being easily withdrawn. Judgment on device success is not required in case of device failure due to inappropriate model/specification selected.<br>②. Success rate of closure<br>"Successful closure from post-operation to discharge": defined as successful deployment of the implanted occluder at the intended target site, with a normal shape, as shown by TTE/TEE, without or with a minimal or small amount of residual shunts (left-to-right shunt signal < 5 mm in diameter based on Doppler left-to-right shunt signals).<br>"Successful closure at 1 month after operation": defined as successful deployment of the implanted occluder at the intended target site, with a normal shape, as shown by TTE/TEE performed at 1 month ± 7 days after operation, without or with a minimal or small amount of residual shunts (left-to-right shunt signal < 5 mm in diameter based on Doppler left-to-right shunt signals).<br>"Successful closure at 3 months after operation": defined as successful deployment of the implanted occluder at the intended target site, with a normal shape, as shown by TTE/TEE performed at 3 months ± 30 days after operation, without or with a minimal or

	small amount of residual shunts (left-to-right shunt signal < 5 mm in diameter based on Doppler left-to-right shunt signals).<br>"Successful closure at 6 months after operation": defined as successful deployment of the implanted occluder at the intended target site, with a normal shape, as shown by TTE/TEE performed at 6 month ± 30 days after operation, without or with a minimal or small amount of residual shunts (left-to-right shunt signal < 5 mm in diameter based on Doppler left-to-right shunt signals).<br>Note: If there is a left-to-right shunt > 5mm, the investigator should determine its clinical significance and cause; successful closure is judged if the shunt is not clinically significant. It is normal if the biodegradable occluder is reduced or irregular (excluding prolapsed or up-and-down lifting) in shape.<br>③. Overall success rate<br>"Overall success" is defined as: successful closure without any severe complications, as shown by TTE/TEE at 6 months ± 30 days after operation.<br>④. Long-term observation<br>"Long-term observation at 12 months after operation": TTE/TEE, relevant laboratory tests and ECG examination will be performed at 12 months ± 30 days after operation to monitor the occurrence of related complications.
Primary Efficacy Endpoint	Evaluation variable: the success rate of closure at 6 months ± 30 days is used as the primary evaluation variable.<br>"Successful closure at 6 months after operation": defined as successful deployment of the implanted occluder at the intended target site, with a normal shape, as shown by TTE/TEE performed at 6 month ± 30 days after operation, without or with a minimal or small amount of residual shunts (left-to-right shunt signal < 5 mm in diameter based on Doppler left-to-right shunt signals).<br>Success rate of closure = (success rate of closure at 6 months after operation/total number of cases) × 100%.
Secondary Efficacy Endpoints	The incidence rates of complications during operation, from post-operation to discharge, as well as at 1 month ± 7 days, 3 months ± 30 days and 6 months ± 30 days after operation are used as secondary evaluation variables.

Schedule of Activities

Test Item	Screening [k]	Surgery	Follow-up				Long-term Observation
Time point			Post-operation to Discharge	1 Month (± 7 days)	3 Months (± 30 days)	6 Months (± 30 days)	12 Months (± 30 days)
Informed consent	X						
Medical history [a]	X						
Demographics [b]	X						
Vital signs [c]	X	X					
Hematology [d]	X			X			X
Urinalysis [e]	X			X	X	X	X
Liver function [f]	X						X
Renal function [g]	X						X
Coagulation function [h]	X						
Blood pregnancy test [i]	X			X	X	X	X
Electrocardiogram [l]	X		X	X	X	X	X
TTE/TEE [m]	X		X	X	X	X	X
ECG monitoring and TTE/TEE monitoring		X					
Inclusion and exclusion criteria	X						
Randomization	X						
ASD closure		X					
Recording of complications		X	X	X	X	X	X
Recording of adverse events and serious adverse events		X	X	X	X	X	X
Medication record [j]	X	X	X	X	X	X	X
