# Supplementary material for: A scaffold-degradable single-rivet ASD occluder with amplatzer-like design: a multicenter randomized controlled trial
Source: Cardiovasc Interv Ther. 2026 Jun 19;41(4):1161–72. doi: 10.1007/s12928-026-01299-7 (PMC13582343; doi:10.1007/s12928-026-01299-7)
Supplement: Supplementary file 7 [file 12928_2026_1299_MOESM7_ESM.pdf]

S.N.	Site	Screening #	Subject Name	Name	Scenario	Severity	Actions Taken	Outcome	Relationship with the Investigational Device	Details of Occurrence and Treatment
1	7	0721	HKDE	Acute tonsillitis; bloodstream infection (infective endocarditis); rash	Leading to hospitalization	Mild	Discontinued in the trial	Resolved (without sequelae)	Not related	Subject HKDE (screening #: BASD-07-21) voluntarily signed the Informed Consent Form (ICF) on 18 Jan 2022 to participate in the "Prospective, Multicenter, Randomized, Controlled Clinical Study on the Efficacy and Safety of Biodegradable ASD Occluder with Delivery Device" sponsored by Shanghai Mallow Medical Device Co., Ltd. The subject was determined to meet all the inclusion criteria but none of the exclusion criteria and enrolled in this study on 18 Jan 2022. DS-12F-850/45 atrial septal defect occluder delivery device and M-BASD-20 occluder were used during the procedure on 19 Jan 2022. The procedure was completed successfully and the subject was discharged on 25 Jan 2022.<br>After discharge, the subject was hospitalized in the Department of Cardiovascular Medicine, Hunan Children's Hospital on 10 Feb 2022 due to recurrent fever. Staphylococcus aureus was detected following multiple blood cultures, and a diagnosis of bloodstream infection (infective endocarditis) was made. On 04 Mar 2022, the subject underwent surgery in the Department of Cardiothoracic Surgery: removal of infective endocarditis vegetation + removal of the occluder + repair of atrial septal defect. The subject was terminated from the trial. The subject was given symptomatic treatment with oxacillin and rifampicin for the infection during hospitalization, and was cured and discharged on 28 Mar 2022. After ethical review, the SAE was considered to be unrelated to the investigational device.<br>On 16 May 2022, the subject visited the Outpatient Department of Cardiovascular Medicine, Hunan Children's Hospital for reexamination 3 months after the atrial septal defect (ASD) occlusion. For the present, the subject is in a generally good condition and fully conscious, with favorable mental reactions, coarse

S.N.	Site	Screening #	Subject Name	Name	Scenario	Severity	Actions Taken	Outcome	Relationship with the Investigational Device	Details of Occurrence and Treatment
										breath sounds in both lungs, without rales, with generally fair heart sounds, regular rhythms without murmurs, and soft abdomen. Laboratory tests: hematology + CRP: neutrophil ratio 0.216 ↓ (NCS); lymphocyte ratio 0.692 ↑ (NCS); mean RBC hemoglobin concentration was 309 ↓ (NCS), and the remaining are all normal. Echocardiography: No obvious transseptal blood color was observed at the repair site of atrial septum; mild mitral and tricuspid regurgitation was observed; left ventricular systolic function was within the normal range. Electrocardiogram: sinus arrhythmia. Chest X-ray: Postoperative changes of congenital heart disease: lung shadow was slightly absorbed than before, heart shadow was slightly enlarged than before, and there was a small amount of pleural effusion to be removed on the right side. Make follow-up clinic visits if any discomfort.<br>The final SAE report was submitted on 17 May 2022
2	7	0728	XWHA	Humeral media epicondyle fracture) (left)	Leading to hospitalization	Mild	Continued in the trial	Symptoms disappeared	Not related	Subject XWHA (screening number: BASD-07-28) voluntarily signed the ICF on 17 May 2022 to participate in the "Prospective, Multicenter, Randomized, Controlled Clinical Study on the Efficacy and Safety of Biodegradable ASD Occluder with Delivery Device" sponsored by Shanghai Mallow Medical Devices Co., Ltd. The subject was determined to meet all the inclusion criteria but none of the exclusion criteria on and enrolled in this trial on 17 May 2022.<br>DS-8F-850/45 atrial septal defect delivery device and M-ASD-12 mm occluder were used during the procedure on 18 May 2022. The procedure was completed successfully. Twenty (20) days ago, the subject fell from a height of 2 meters with the left elbow touching the ground, feeling local pain, without bleeding, skin damage or fever, and gradually developed elbow swelling that resulted in limited activity. The

S.N.	Site	Screening #	Subject Name	Name	Scenario	Severity	Actions Taken	Outcome	Relationship with the Investigational Device	Details of Occurrence and Treatment
										subject was diagnosed with "dislocation of left elbow joint" in Lianyuan Coal Hospital and given fixation with plaster for 3 weeks. Before the inpatient surgery at the Department of Cardiovascular Medicine, the external fixation of elbow joint had been removed for about 1 week. X-ray on 21 May 2022 reported: 1. Local fracture of left medial distal humerus. 2. Small strip-like bone density shadow at the lateral side of the left distal humerus, possible avulsion fracture. Orthopedics consultation: 1. Perform elbow CT plain scan (three-dimensional imaging); anteroposterior and lateral X-rays of the right elbow; 2. The subjects' elbow joint is stiff and the flexion and extension activities are obviously limited. Make functional exercise, and undergo a surgical procedure after full healing of the fracture, if necessary, when the function fails to recover fully. 3. Make a follow-up visit to the orthopedics department for re-examination after the cardiovascular disease has improved. Examinations were performed on 22 May 2022: X-ray: anteroposterior and lateral views showed no obvious abnormality of the right elbow. CT: Fracture of the left distal humerus, no dislocation of the left elbow. On 23 May 2022, the subject was discharged and made a follow-up clinic visit to the orthopedics department, where inpatient treatment and anesthesiology consultation were recommended. Anesthesiology consultation: The subject is currently taking aspirin, which may affect platelet function and cause intraoperative massive hemorrhage. In addition, the subject should avoid strenuous exercise for about half a year. Surgical anesthesia may cause displacement of the occluder, and anesthetic risks have been told to the subject's family.<br>On the afternoon of 23 May 2022, the patient was admitted to Orthopedics Department, Hunan Children's

S.N.	Site	Screening #	Subject Name	Name	Scenario	Severity	Actions Taken	Outcome	Relationship with the Investigational Device	Details of Occurrence and Treatment
										Hospital. Physical examination reported no abnormality. Hematology showed red blood cell count (RBC): 4.13 * 10^12/L ↓, and the remaining are all normal. The physician diagnosed "1. humeral media epicondyle fracture (left) 2. recovery period after surgery (after ASD surgery (ostium secundum type))" and concluded probable stiffness of the elbow where the fixation was performed. The subject was advised to continue the functional exercise of elbow flexion and extension, and to make a follow-up visit for re-examination 2 months later to determine the necessity of surgical treatment. The subject was discharged on 24 May 2022.<br>Initial SAE report was submitted on 24 May 2022.<br>On 08 Aug 2022, the subject came to Hunan Children's Hospital for 3-month follow-up after atrial septal defect closure, saying that he or she was diagnosed as old left humeral media epicondyle fracture: medial protrusion of the left elbow, without tenderness, extension 0-130°, and normal activities of left fingers on 04 Aug 2022 after a follow-up visit to Hunan Children's Hospital for reexamination due to "humeral media epicondyle fracture (left)". Outpatient doctor's recommendations: 1. active elbow flexion and extension training; 2. temporarily avoid strenuous exercise; 3. make a clinic visit for re-examination 2 months later； and 4. make a clinic visit if any discomfort. Examinations performed today: ECG and urinalysis + urine sediment results were generally normal; echocardiography: 1. No obvious transseptal blood flow observed around the ASD occluder. 2. The ventricular systolic function is within the normal range. Physical examination: clear breath sounds in both lungs, fair heart sounds, no murmurs, and soft abdomen.<br>A follow-up report for the SAE was submitted on 08 Aug 2022.

S.N.	Site	Screening #	Subject Name	Name	Scenario	Severity	Actions Taken	Outcome	Relationship with the Investigational Device	Details of Occurrence and Treatment
										On 21 Nov 2022, the subject came to Hunan Children's Hospital for 6-month follow-up after transcatheter ASD closure. Examinations performed today: normal ECG; urinalysis + urine sediment: occult blood +-(NCS), and the remaining are all generally normal; echocardiography: 1. No obvious transseptal blood flow observed around the ASD occluder. 2. The left ventricular systolic function is within the normal range. Physical examination: clear breath sounds in both lungs, fair heart sounds, no murmur, and soft abdomen. The parent of the subject complained that repeated X-ray in Lianyuan Coal Hospital on 20 Aug 2022 showed slight deformation of the medial hand joint, and the doctor assessed that it would not affect bone healing and no further re-examination is necessary since the subject's elbow flexion and extension activities are not significantly limited. Therefore, the final report is submitted today.
3	7	0738	ZRNI	Pyrexia	Prolonging hospitalization	Mild	Continued in the trial	Resolved without sequelae	Not related	Subject ZRNI (screening number: BASD-07-38) voluntarily signed the ICF on 11 Oct 2023 to participate in the "Prospective, Multicenter, Randomized, Controlled Clinical Study on the Efficacy and Safety of Biodegradable ASD Occluder with Delivery Device" sponsored by Shanghai Mallow Medical Device Co., Ltd. The subject was determined to meet all the inclusion criteria but none of the exclusion criteria and enrolled in this trial on 12 Oct 2023.<br>M-DS-8F-850/45 atrial septal defect delivery device and M-ASD-12 mm occluder were used during the procedure on 12 Oct 2023. The procedure was completed successfully. After the procedure, aspirin enteric-coated tablets 50 mg QD po was given for anti-platelet aggregation. Postoperative echocardiography showed that the occluder was normal in position and shape. ECG showed no conduction

S.N.	Site	Screening #	Subject Name	Name	Scenario	Severity	Actions Taken	Outcome	Relationship with the Investigational Device	Details of Occurrence and Treatment
										block. The subject developed a fever on 14 Oct 2023 and was given oral Ibuprofen Suspension for bringing down the fever. The subject had the complication of cough later, and relevant examinations were performed, showing significantly increased serum amyloid protein, for which bacterial infection was considered. Cefuroxime Sodium (0.3 g q8h from 15 Oct 2023 to 19 Oct 2023) for anti-infection and Compound Pholcodine Oral Solution 5 mL po Tid for relieving cough were given. On 19 Oct 2023, the subject had no chest distress, chest pain, fever or cough, and was in good spirits, without abnormality of urine or stool. The subject's hospitalization was prolonged today, and the AE (fever) turned into an SAE.<br>On 20 Oct 2023, the subject had no chest distress, chest pain, no fever, no cough, and was in good spirits without abnormality of urine or stool. With an improved condition, the subject is discharged today.<br>On 14 Nov 2023, the subject visited the hospital for 1-month follow-up after operation: urinalysis + urinary sediment: vitamin C (VC) 2 + (NCS), and the remaining are all normal. Hematology: eosinophil ratio (EO): 0.155, ratio ↑ (NCS), and the remaining are all normal. ECG: 1. Sinus rhythm; 2. Complete right bundle branch block (NCS). Echocardiography: one month after atrial septal defect occlusion (metal type): 1. No obvious transseptal blood color observed around the ASD occluder. 2. The left ventricular systolic function is within the normal range. The guardian of the subject complained that the subject had no fever, cough, chest distress, chest pain or other symptoms since discharge, with a general condition, without experiencing any new AE, SAE or complications.<br>The SAE follow-up + final report was submitted on 14 Nov 2023.

S.N.	Site	Screening #	Subject Name	Name	Scenario	Severity	Actions Taken	Outcome	Relationship with the Investigational Device	Details of Occurrence and Treatment
4	11	1110	ZHJU	Thyroid neoplasm malignant	Leading to hospitalization	Severe	Continued in the trial	Resolved with sequelae	Not related	A 48-year-old female patient ZHJU (ethnic group: Han) signed the ICF on 27 Mar 2023 to voluntarily participate in the "Prospective, Multicenter, Randomized Controlled, Clinical Study on the Efficacy and Safety of Biodegradable ASD Occluder with Delivery Device" (protocol No.: JK-LC-BASD-01), and was then assigned a screening number: BASD-11-10. On 27 Mar 2023, relevant examinations/tests were performed according to the requirements of the clinical study protocol, showing that the patient met all the inclusion criteria but none of the exclusion criteria. The patient was enrolled in the clinical trial of the biodegradable ASD occluder on 28 Mar 2023. On 28 Mar 2023, the patient underwent "closure of atrial septal defect of congenital heart disease". The patient completed the follow-up from post-operation to discharge on 30 Mar 2023.<br>The investigator was informed on 13 Oct 2023 that the subject underwent ultrasonography of the neck 3 months ago on 27 Jul 2023 and was shown to have "goiters" without redness, swelling or pain. Ultrasonography repeated in another hospital showed "nodules in the right lobe of thyroid, suspected to be malignant". No other diagnosis or treatment was given, and the patient was admitted to the outpatient department of Linyi Cancer Hospital due to "goiters, malignant?". Relevant examinations/tests were performed after admission. Since there was no surgical contraindication, the patient underwent radical thyroidectomy (isthmus thyroidectomy, left lobectomy, partial right lobectomy, anterior laryngeal and left central neck dissection, recurrent laryngeal nerve exploration) under general anesthesia on 01 Aug 2023. The surgical procedure was completed successfully, and the patient recovered well after postoperative supportive treatment and was

S.N.	Site	Screening #	Subject Name	Name	Scenario	Severity	Actions Taken	Outcome	Relationship with the Investigational Device	Details of Occurrence and Treatment
										discharged. Postoperative pathology: (left lobe) papillary thyroid microcarcinoma, tumor diameter: 0.5 cm, with nodular goiter changes in surrounding tissues; no cancer identified in one lymph node in front of the larynx and a few thyroid tissues, and 2 lymph nodes in VI region of the left neck; nodular goiter (right lobe). On 05 Aug 2023, the patient was discharged with a discharge diagnosis of " thyroid cancer T1NOMO and hypertension after atrial septal defect closure". After discharge, the patient followed the doctor's advice "take Levothyroxine Tablets 50 μg, po, qd; return to the hospital one month later for fasting blood draw for a repeated thyroid function test, and if necessary, adjust the dosage of Levothyroxine Tablets depending on the test result".<br>On 09 Oct 2023, the subject was re-examined for "thyroid function" in Linyi Cancer Hospital, without abnormality. The dosage of Levothyroxine was not adjusted.<br>The investigator understood the current situation of the subject and paid close attention to the subsequent treatment of the subject. The initial follow-up report for the SAE "thyroid malignancy" is reported today, concluding that the SAE was CTCAE "Grade 3" and not related to the investigational medical device. Whether this SAE experienced by the subject will affect the subsequent study observation and results needs to be determined through further close observation.<br>On 05 Dec 2023, the subject underwent a repeat "thyroid function test" in Linyi Cancer Hospital, which reported "T3: 0.81 ↓, TSH: 12.72 ↑, and CS hypothyroidism." The dosage of levothyroxine was adjusted to "Levothyroxine Tablets 50 μg, po, bid, 1 tablet each time".<br>On 11 Apr 2024, the subject underwent a repeat "thyroid

S.N.	Site	Screening #	Subject Name	Name	Scenario	Severity	Actions Taken	Outcome	Relationship with the Investigational Device	Details of Occurrence and Treatment
										function test" in Linyi Cancer Hospital, which reported "T3: 0.68 ↓, TSH: 11.25 ↑, thyroglobulin 0.767 ↓, and CS hypothyroidism." The dosage of levothyroxine was adjusted to "Levothyroxine Tablets 50 μg, po, bid, two and a half tablets each time". On 05 Dec 2023, the dosage of levothyroxine was adjusted as "Levothyroxine Tablets 50 μg, po, bid, 2 tablets each time". The subject is a patient with tumor and received re-treatment of the underlying disease, which was not actually related to the investigational device involved in this project or to the complications associated with the investigational device. Close attention in future is recommended. The SAE final report for the event of "thyroid malignancy" is submitted today.
**5**	13	1320	WALI	Early pregnancy termination	Leading to hospitalization	Mild	Continued in the trial	Resolved without sequelae	Not related	A 35-year-old female patient WALI (ethnic group: Han) signed the ICF on 08 Aug 2023 to voluntarily participate in the "Prospective, Multicenter, Randomized Controlled, Clinical Study on the Efficacy and Safety of Biodegradable ASD Occluder with Delivery Device" (protocol No.: JK-LC-BASD-01), and was then assigned a screening number: BASD-13-020. On 08 Aug 2023, relevant examinations/tests were performed according to the requirements of the clinical study protocol, showing that the patient met all the inclusion criteria but none of the exclusion criteria. The patient was enrolled in the clinical trial of the biodegradable ASD occluder on 10 Aug 2023. On 10 Aug 2023, the patient underwent "closure of atrial septal defect of congenital heart disease". The patient completed the follow-up from post-operation to discharge on 11 Aug 2023.<br>On 31 Aug 2023, the investigator was informed that the subject felt discomfort due to nausea and vomiting on 30 Aug 2023, with an abnormal physiological period, and had a positive result of urine pregnancy test using test

S.N.	Site	Screening #	Subject Name	Name	Scenario	Severity	Actions Taken	Outcome	Relationship with the Investigational Device	Details of Occurrence and Treatment
										strip on her own. On 30 Aug 2023, the subject visited the Eighth Hospital of Wuhan for transvaginal ultrasound examination, which suggested early intrauterine pregnancy, with an ultrasound gestational age of 4 weeks and 2 days; a small amount of pelvic effusion. Considering the post-operative use of anti-platelet aggregation agents, the subject decided to terminate the pregnancy. The Eighth Hospital of Wuhan recommended hospitalization for relevant examinations/tests to facilitate subsequent treatment. The patient orally took Mifepristone 4 tablets QD on 30 Aug 2023 and Mifepristone 4 tablets, QD on 31 Aug 2023. Induced pregnancy termination with drug was performed.<br>The patient visited the hospital on 12 Sep 2023 to complete the follow-up one month after operation. The follow-up information on the SAE "early pregnancy termination" is updated as follows:<br>The patient experienced vaginal bleeding equal to the menstrual volume following oral administration of Misoprostol on the noon of 01 Sep 2023. The patient complained of tissue discharge that was flushed into the toilet. In the afternoon, the patient underwent painless artificial abortion under B ultrasound monitoring. Preoperative B ultrasound suggested no intrauterine echo of gestational sac, about 30 g of tissue was sucked and scraped, without typical villi. The procedure was completed smoothly, and postoperative uterine contraction was good. Postoperative B ultrasound showed no intrauterine abnormal echo, with little vaginal bleeding. Anti-inflammatory symptomatic treatment (clindamycin, metronidazole) was prescribed for the patient (details about the dosage were not known). Postoperative pathological examination showed: blood clots (intrauterine pregnancy tissue), decorticated tissue and a few trophoblast cells were

S.N.	Site	Screening #	Subject Name	Name	Scenario	Severity	Actions Taken	Outcome	Relationship with the Investigational Device	Details of Occurrence and Treatment
										observed under microscope. On the morning of 05 Sep, the patient was informed of pathological examination results by phone, asking the patient to come to the hospital for a repeat blood HCG test, but the patient refused. On 12 Sep 2023, repeat blood HCG test showed a result of 142.181 U/L. The SAE is ongoing, and close attention will be paid to the subject's subsequent treatment. A follow-up report for the SAE "early pregnancy termination" was reported, concluding that the event was mild and not related to the investigational medical device.<br>Added the AE of "vaginitis", start date: 30 Aug 2023, paroxysmal, number of seizures: 1; severity: mild. The patient was given symptomatic treatment with clindamycin and metronidazole (details about the dosage were not known). The symptoms were resolved on 12 Sep 2023, and the AE ended. It was not considered as SAE. The patient did not withdraw from the trial due to the AE. The AE was not related to the investigational device. Possibly related to the SAE.<br>On 21 Nov 2023, the patient visited the hospital to complete the 3-month follow-up after operation, and the follow-up information on the SAE "early pregnancy termination" is updated as follows:<br>In the initial report, the subject decided to terminate the pregnancy considering the administration of anti-platelet aggregation agents after operation. After confirmation with the patient through multiple communications, the information is corrected to the following: the patient has two children and is voluntarily determined to terminate the pregnancy. Blood HCG test repeated on 21 Nov 2023 showed a normal result of < 2.30 IU/L. The SAE ended on 21 Nov 2023, and the final report for the SAE "early pregnancy termination" is submitted today, concluding that it was not related to the investigational medical

S.N.	Site	Screening #	Subject Name	Name	Scenario	Severity	Actions Taken	Outcome	Relationship with the Investigational Device	Details of Occurrence and Treatment
										device.
**6**	1	0107	XXRO	Foreign body in left atrium	Leading to hospitalization	Moderate	Discontinued in the trial	Resolved (without sequelae)	It may not be relevant	The patient XXRO was admitted to the hospital due to the diagnosis of "atrial septal defect" on August 14, 2022. The patient and his legal guardian learned about the prospective, multicenter, randomized controlled clinical trial of "effectiveness and safety of degradable ASD occluder and delivery device" sponsored by Shanghai kumquat Medical Devices Co., Ltd. on August 14, 2022. After full consideration and understanding of the clinical project, the patient and his legal guardian<br>The informed consent was signed on August 15, and the screening number was basd-01-07. According to the trial<br>According to the inspection plan, the relevant tests were collected and improved during the screening period. The diameter of the atrial septal defect was 6.0*8.6mm according to the ultrasound. According to the inspection results, the investigator judged that it met the inclusion criteria of this trial, and it did not meet the exclusion criteria of this trial. It was selected into this trial, and it was entered into the experimental group according to the content of the random letter.<br>The subject underwent percutaneous cardiac catheterization +asd closure on August 16, 2022, and the test device m-basd-12 occluder was used intraoperatively [serial number:<br>121912001093]. Intraoperative observation, the test instrument was released successfully at one time, and ultrasound<br>And the angiogram showed that the position was ok, the morphology was normal, there was no residual shunt, and there was no obvious atrioventricular valve regurgitation<br>Flow. During the whole operation, ECG monitoring showed that there was no arrhythmia and the operation was successful. Postoperative medication

S.N.	Site	Screening #	Subject Name	Name	Scenario	Severity	Actions Taken	Outcome	Relationship with the Investigational Device	Details of Occurrence and Treatment
										Aspirin enteric coated tablets 100mg QD orally, clopidogrel bisulfate tablets 20mg<br>Oral anticoagulation. On August 19, 2022, the subject had no postoperative discomfort and postoperative injury<br>The mouth recovered well, the dorsalis pedis artery pulsation was symmetrical, and the urine color was clear, so they were discharged to the outpatient clinic for follow-up.<br>Take aspirin enteric coated tablets 100mg orally QD for half a year and clopidogrel bisulfate tablets 20mg orally QD after discharge.<br>On September 15, 2022, he came to the hospital for 1-month follow-up examination after operation, and the skin<br>There was bruising on the skin without other discomfort, and the ultrasound examination showed that "there was stuffing along the level of the atrial septum<br>The device and occluder are in normal position and shape, and no residual points are measured around it<br>Flow. ”Record the AE events of skin bruising, adjust anticoagulant medication, and take the aspirin<br>Aspirin enteric coated tablets were adjusted to 50mg, clopidogrel sulfate tablets to 12.5mg,<br>Follow up if you feel unwell.<br>On November 3, 2022, the patient was followed up for 3 months after operation, and there was no chief complaint of discomfort.<br>The ultrasonic examination showed that "the tamponade device was seen along the level of the atrial septum, and the position of the occluder was normal,<br>The morphology was normal, and no residual shunt was detected along its periphery. ”AE event skin bruising<br>The situation still occurred. The anticoagulant medication was adjusted again, and the aspirin enteric

S.N.	Site	Screening #	Subject Name	Name	Scenario	Severity	Actions Taken	Outcome	Relationship with the Investigational Device	Details of Occurrence and Treatment
										coated tablets were adjusted<br>The dosage of clopidogrel sulfate tablets remained unchanged at 12.5mg, and the patients were advised to follow up with their discomfort<br>Diagnosis.<br>On February 2, 2023, the patient was followed up for 6 months after operation, and there was no chief complaint of discomfort. Ultrasound examination showed that "the tamponade device was seen along the level of the atrial septum, the occluder position was normal, the shape was normal, and no residual shunt was detected along its periphery." the dosage of aspirin enteric coated tablets 25mg and clopidogrel sulfate tablets remained unchanged at 12.5mg, and follow-up was ordered for discomfort.<br>The subject came to the hospital on May 4, 2023 and June 15, 2023 for follow-up due to occasional headache and skin bruising. The ultrasound examination showed that "the level of the atrial septum can see the tamponade device, the position of the occluder is normal, the left atrial disc can be seen floating, the thin filament residual shunt is measured along its upper edge, the bundle width is 1.0mm, and the left to right shunt. Continue the previous anti platelet aggregation and anti platelet aggregation drug regimen, and closely observe,<br>Follow up for discomfort.<br>The subject still had occasional headache, and slight collision of both lower extremities would cause skin bruising. The investigator suggested admission for observation. The subject was hospitalized on 2023<br>On July 3, 2004, he was admitted to the hospital, and the ultrasound examination showed that "the tamponade device was seen along the level of the interatrial septum, the position of the occluder was normal, and the tissue fluttered on the left atrial side, the size of

S.N.	Site	Screening #	Subject Name	Name	Scenario	Severity	Actions Taken	Outcome	Relationship with the Investigational Device	Details of Occurrence and Treatment
										6.2mm*11.0mm, There was no significant residual shunt. ”Further inspection is recommended.<br>The 2023-07-05 MRI report suggested that the child was "after atrial septal defect intervention", and there was no obvious residual shunt; Left ventricular systolic and diastolic function is normal, please combine clinical and ultrasound examination. No obvious abnormality was found in delayed left ventricular enhancement scan. According to the medical history, physical examination and auxiliary examination, the investigator found that the subject was at risk due to unknown foreign body in the left atrium, which required surgical treatment.<br>After the subject was admitted to the hospital, the relevant examinations were improved, and the contraindications to surgery were excluded. On July 5, 2023, general anesthesia was performed. 1. repair of atrial septal defect; 2. during the removal of the plugging device, the following were seen: routine disinfection and towel laying, under aerostatic anesthesia, the right axillary straight incision, the right umbrella disc contour of the plugging device can be seen at the position of the foramen ovale of the atrial septum, about 4 × 6mm, scar like, the atrial septum was cut along the umbrella disc, no obvious umbrella disc contour was found on the left atrial surface, but there was a scar cord attached, about 3mm in diameter, the end expanded, like the scar tissue formed after the degradation of the plugging device, about 9mm in diameter, free in the left atrium. The closure device and a small amount of atrial septal tissue were excised, the atrial septal defect was repaired with continuous suture of pericardial patch, and the patient was returned to ICU with endotracheal intubation. The material was removed for pathological analysis. The subject terminated the trial.<br>On July 5, 2023, the subject's postoperative blood

S.N.	Site	Screening #	Subject Name	Name	Scenario	Severity	Actions Taken	Outcome	Relationship with the Investigational Device	Details of Occurrence and Treatment
										pressure was low, albumin was given to supplement blood volume, dopamine and milrinone were continued to maintain circulation stability, compound chlorhexidine containing solution was given for antibacterial treatment, concentrated sodium chloride injection was given, 10% sodium chloride injection was given to supplement electrolyte, midazolam injection was given, dexmedetomidine hydrochloride injection was given for sedation, cefuroxime sodium for injection was given for anti infection, ibuprofen suspension was given for antipyretic treatment, dexamethasone sodium phosphate injection was given for anti-inflammatory treatment, budesonide suspension for inhalation was given for anti-inflammatory and antiasthmatic treatment, the subject was conscious, spontaneous breathing was active, sputum aspiration had cough cough reflex, and the ventilator was evacuated and nasal tube was changed for oxygen inhalation.<br>On July 6, 2023, the subject was oxygenated with oxygen through both nasal passages, and had fever, temperature of 38.6 ℃, no cough, asthma, diarrhea, vomiting and stomach intake twice last night. The amount of intake was 1037ml, the amount of output was 1164ml, and the chest catheter was 85ml. ECG monitoring:<br>Heart rate 99 beats / min, blood pressure 98 / 57mmhg, respiration 22 beats / min, blood oxygen saturation 100%. The children continued to receive dopamine to maintain circulation stability, cefuroxime to fight infection, ibuprofen suspension to reduce fever, 10% sodium chloride injection, and concentrated sodium chloride injection to supplement electrolytes. Auxiliary examination: cardiac ultrasound (in infant heart<br>Xin: [examination result]after removal of ASD degradable device and repair of atrial septum: no

S.N.	Site	Screening #	Subject Name	Name	Scenario	Severity	Actions Taken	Outcome	Relationship with the Investigational Device	Details of Occurrence and Treatment
										obvious residual shunt.<br>On July 7, 2023, the subject was oxygenated by giving oxygen to both nasal passages, but his temperature still fluctuated, 38.0 ℃, no cough, asthma, diarrhea, 1440ml yesterday, 1430ml output, 25ml chest catheter. ECG monitoring: heart rate 110 beats / min, blood pressure 92 / 60mmhg, 22 breaths / min, blood oxygen saturation 100%.<br>The subject is currently able to maintain postoperative oxygenation, maintain stable circulation, stop vasoactive drugs, stop sedatives, and continue cefuroxime anti infection, potassium citrate oral solution diuresis and potassium supplement treatment.<br>On July 8, 2023, the subject was oxygenated by giving oxygen through both nasal passages, and there was no high fever. The body temperature gradually tended to be normal, without cough, asthma, diarrhea, and mental stomach. The subject stopped oxygen inhalation today, adjusted the frequency of furosemide, continued cefuroxime anti infection, potassium citrate oral solution diuresis and potassium supplement treatment, and removed the chest catheter.<br>On July 9, 2023, the subject maintained oxygen saturation under nasal tube oxygen inhalation,<br>No fever, cough and asthma, diarrhea, and psycho gastric intake. Continue to give cefuroxime anti<br>Infection, atomization, diuresis, potassium supplement and other symptomatic support treatment.<br>On July 10, 2023, the subject maintained oxygen saturation under nasal tube oxygen inhalation,<br>No fever, cough and asthma, diarrhea, and psycho gastric intake. Continue cefuroxime anti infection, potassium citrate oral solution diuresis potassium supplement treatment. Pathological analysis results of the removed objects: foreign bodies with multinucleated

S.N.	Site	Screening #	Subject Name	Name	Scenario	Severity	Actions Taken	Outcome	Relationship with the Investigational Device	Details of Occurrence and Treatment
										giant cell reaction, hyperplasia of fibrous and granulation tissues, scattered chronic inflammatory cell infiltration were found in the submitted objects, and no obvious abnormalities were found in the rest.<br>On July 11, 2023, the oxygen saturation of the subject was maintained without oxygen inhalation,<br>No fever, no cough and wheeze, no vomiting and diarrhea, good mental appetite, no difference between two stools. Remove the deep vein catheter. Now the subject's general condition is acceptable, and the postoperative reexamination has been improved. The cardiac ultrasound has no difference, so he was discharged from the hospital.<br>According to the analysis of the left atrial extract and pathological results, it can be seen that the subject<br>The contour of the right umbrella disc of the implanted degradable occluder device, the mesh on the right side of the occluder device has been basically degraded, and the intracardiac tissue has climbed and grown smoothly; The left reticular body had been completely degraded, and the intracardiac tissue climbed and grew abnormally, attached to the atrial septum in a strip shape, and the end was expanded; The foreign body in the left atrium removed by this operation may be related to granulation tissue hyperplasia and abnormal growth, and may not be related to the test equipment.<br>On September 25, 2023, the subject came to our hospital for postoperative follow-up, and there was no discomfort<br>Complaint, ECG: normal range ECG, echocardiography: ASD degradable device removal and atrial septum repair: no obvious residual shunt. On October 2023<br>Report SAE summary report on November 11.
7	3	0311	ZXZH	Left basal ganglia hemorrhag	Leading to hospitalization	Moderate	Continued in the trial	Resolved without sequelae	Not related	Screening number: basd-03-11 subject zxzh, female, 55 years old, Han nationality, signed the informed consent on May 16, 2023, indicating that he voluntarily

S.N.	Site	Screening #	Subject Name	Name	Scenario	Severity	Actions Taken	Outcome	Relationship with the Investigational Device	Details of Occurrence and Treatment
				e ruptured into ventricle						participated in the "prospective, multicenter, randomized controlled clinical trial of the effectiveness and safety of degradable ASD occluder and delivery device" (protocol number: jk-lc-basd-01). The investigator performed relevant examinations on the subjects according to the requirements of the clinical protocol. According to the examination results, it was determined that the subjects met the inclusion criteria of this trial and did not meet any exclusion criteria. They were enrolled in the clinical trial of degradable ASD occluder on May 16, 2023, and entered the control group according to the contents of No. 03010 random envelope. The subject underwent "congenital heart ward septal defect closure" on May 17, 2023, and the operation was successful. The investigator observed that the subject was in good condition without special discomfort on May 18, 2023. The ultrasound report from postoperative to discharge showed that the atrial horizontal shunt disappeared after the closure of atrial septal defect. The subject was discharged on May 18, 2023. The subject was followed up according to the clinical protocol at 1 month, 3 months and 6 months after operation, and there were no symptoms of discomfort and no new adverse events. The combination of aspirin was ended on November 16, 2023.<br>The investigator communicated with the subject by telephone on May 31, 2024 to make an appointment for a one-year visit after the operation. The subject complained that he was admitted to the local Xing'an League people's hospital due to sudden unconsciousness on December 2, 2023. After the relevant examination, the diagnosis was that the left basal ganglia hemorrhage had broken into the brain ventricle. After the relevant examination was improved, the intracerebral hematoma was cleared and decompressed, and the operation was successful. After the operation, the patients were given

S.N.	Site	Screening #	Subject Name	Name	Scenario	Severity	Actions Taken	Outcome	Relationship with the Investigational Device	Details of Occurrence and Treatment
										bromhexine hydrochloride injection for expectorant, acetylcysteine solution for inhalation, glycerol fructose sodium chloride injection for rehydration, glucose injection, mannitol injection for reducing intracranial pressure, cefuroxime sodium for anti-inflammatory and symptomatic treatment injection, piperacillin sodium and tazobactam sodium for injection, and the clearance effect of cranial CT was satisfactory after the operation. The subject developed aggravated cough and expectoration on December 7, 2023, with a large amount of yellow sputum, accompanied by dyspnea. On December 8, 2023, he underwent tracheotomy under general anesthesia. After symptomatic treatment, spontaneous breathing and expectoration improved, tracheal cannula was removed, and vital signs were stable. On January 8, 2024, his condition improved and he was discharged. The subject complained about the sequelae of limited limb movement and limited walking caused by the SAE event, and was unable to return to our hospital for a 1-year follow-up visit in accordance with the requirements of the plan. The subject was examined in the local hospital. The investigator expressed understanding and asked the subject to send the examination results to the investigator for observation and judgment. The ultrasound examination results and electrocardiogram results were normal.<br>This serious adverse event is not actually related to the test device involved in this clinical trial project and the related complications involved in the test device. The investigator judged that it is definitely not related to the test device, and reported the SAE first report and summary report today.
**8**	**3**	0316	ZZHA	Acute drug poisoning	Leading to hospitalization	Moderate	Continued in the trial	Resolved without sequelae	Not related	The patient ZZHA, female, 15 years old, Han nationality, signed the informed consent on August 8, 2023, indicating that she voluntarily participated in the

S.N.	Site	Screening #	Subject Name	Name	Scenario	Severity	Actions Taken	Outcome	Relationship with the Investigational Device	Details of Occurrence and Treatment
										"prospective, multicenter, randomized controlled clinical trial of the effectiveness and safety of degradable ASD occluder and delivery device" (protocol number: jk-lc-basd-01), and subsequently obtained the screening number basd-03-16. The relevant examinations were performed according to the requirements of the clinical protocol on August 8, 2023. According to the examination results, the investigator judged that it met the inclusion criteria of this trial, but did not meet any exclusion criteria. The patient and his family members who collected the medical history reported that they had depression and anxiety in the past, took oral medication, and their symptoms improved. At the time of enrollment, the patient was in stable mood, in good condition, and enrolled on August 8, 2023 degradable ASD In the clinical trial of the occluder, the "closure of congenital heart ward septal defect" was performed on August 9, 2023. The patient was generally in good condition without special discomfort on August 10, 2024. The follow-up from postoperative to pre discharge was improved. The ultrasound report from postoperative to pre discharge showed that after the closure of atrial septal defect, the atrial horizontal shunt disappeared. After informing the discharge precautions, the discharge procedures were handled.<br>During the self-examination of the project on May 13, 2024, the investigator found that the subject was admitted to the hospital for examination due to oral overdose on March 8, 2024, and did not timely inform the investigator that the drugs taken were: aripiprazole 5mg/ tablet, about 5 tablets, tandospirone citrate capsules 5mg/ tablet, 24 tablets left and right, mirtazapine tablets 30mg/ tablet, about 3 tablets, venlafaxine hydrochloride sustained-release capsules 7mg/ tablet, about 8 tablets. After taking the medicine,

S.N.	Site	Screening #	Subject Name	Name	Scenario	Severity	Actions Taken	Outcome	Relationship with the Investigational Device	Details of Occurrence and Treatment
										the child had nausea, no vomiting, obvious sleepiness, occasional upper limb twitching, and no abnormal increase in muscle tone. The emergency department of the first hospital of Jilin University received the "drug poisoning" into the pediatric ICU department. After gastric lavage, fluid replacement and drainage promotion, blood perfusion and related symptomatic support treatment, the subject's symptoms gradually improved, and the diagnosis of acute drug poisoning and depression was made again. On March 15, 2024, the patient's condition was good, his vital signs were stable, and he had reached the discharge standard. After informing the discharge precautions, he was given the discharge procedures. The serious adverse event has no actual correlation with the test device involved in the clinical trial project and the related complications involved in the test device, and the investigator judged that it has nothing to do with the test device.<br>The investigator learned by telephone that the subject has not experienced any other discomfort since he was discharged from the hospital on March 15, 2024. Follow up closely and report the SAE first report and summary report today.
**9**	7	0701	JYNA	1. headache (Chayin) 1.1 cerebral infarction (?) 1.2 cerebral hemorrhage 1.3 cerebral white matter (?)	Leading to hospitalization	Mild	Continued in the trial	Resolved without sequelae	Not related	Screening number BASD-07-01 Subject JYNA voluntarily signed an informed consent form on May 31, 2021 to participate in the "Biodegradable ASD Occluder and Delivery Device Effectiveness and Safety" sponsored by Shanghai Mallow Medical Devices Co., Ltd. Prospective, multi-center, randomized controlled "clinical trial met the selection criteria for this trial on June 2, 2021, and did not meet the exclusion criteria for this trial, and was selected for this trial. On June 2, 2021, the DS-10F-850/45 heart defect occluder delivery device and M-MF-BASD-2424 specification occluder were used during the operation, and the operation went

S.N.	Site	Screening #	Subject Name	Name	Scenario	Severity	Actions Taken	Outcome	Relationship with the Investigational Device	Details of Occurrence and Treatment
				2. meniscus injury						smoothly. The subject was cured and discharged on June 5, 2021. On May 31, 2021, the DS-10F-850/45 heart defect occluder delivery device and M-MF-BASD-2024 specification occluder were used during the operation, and the operation went smoothly. The general condition of the subjects after surgery was good. On June 3, 2021, the heart color Doppler ultrasound showed: 1. There was no obvious septal blood color around the atrial septal occluder. 2 Normal range of cardiac systolic function. On June 5, 2021, the subject was cured and discharged with medicine: aspirin enteric-coated tablets 100mg orally once a day (half a year) (self-provided). One month after the operation of the subject on July 5, 2021, three months after the operation of the subject on August 30, 2021, and six months after the operation of the subject on December 13, 2021, the heart color Doppler ultrasound of the subject was "atrial septal occluder The shape is normal, the position is normal, and there is no obvious septal blood color around. 2. The left heart systolic function is normal." The subject developed headache after exertion on February 11, 2022, paroxysmal, gradually aggravated from morning to bedtime, and could not relieve itself; On February 13, 2022, the headache disappeared the next day after taking "aspirin 100mg/tablets 2 tablets" by myself; On February 14, 2022, I developed pain during walking and compression on the left calf. On the same day, I went to the Second Affiliated Hospital of Nanchang University to improve the MRI findings: "1. There are few ischemic foci in the white matter of the bilateral frontal lobes. 2. No obvious abnormalities in MRA." For further diagnosis and treatment, the subjects came to Hunan Children's Hospital for treatment on February 15, 2022, and the outpatient department was admitted to the hospital for treatment with "headache investigation".

S.N.	Site	Screening #	Subject Name	Name	Scenario	Severity	Actions Taken	Outcome	Relationship with the Investigational Device	Details of Occurrence and Treatment
										The subjects had occasional falls on the flat ground, dizziness after standing up suddenly, blackness in front of the eyes, knee joint pain (frequency 2-3 times a month), etc., and had good mental intake, normal urination and defecation, and no weight loss. At 18:05 pm on February 15, 2022, Dr. Zeng Min checked the subjects, Preliminary diagnosis: headache Chain: 1. Headache (Chain) 1.1 Cerebral infarction (?): Subject Patients had headache, a history of cerebral embolism in the past, and MRI findings in other hospitals: 1. Bilateral frontal A little ischemic focus in leaf white matter 2. MRA showed no obvious abnormalities. If you have a recurrent headache, you need to Be vigilant. 1.2 Cerebral hemorrhage): The subject had headache symptoms and previous cerebral infarction Cerebral CT was performed to make a definite diagnosis. 1.3 Leukoencephalopathy): MRI in the external hospital of the child Suggestions: 1. A few ischemic foci in bilateral frontal white matter 2. No obvious abnormalities were found in MRA, Combined with the subject's clinical manifestations and past medical history, we need to be vigilant. 2. Intervention of atrial septal defect After closure: The subject underwent interventional closure of atrial septal defect in our hospital in 2021 Art. 3. Meniscus injury): Subjects are swimmers, active, Private complaints of previous knee pain, the frequency is about 2-3 times in January. At 10:00 am on February 16, 2022, Dr. Zeng Min checked the subjects, The subjects had dizziness, good diet and sleep, and normal defecation and urination. The spirit can be, but no skin Rash, subcutaneous hemorrhage, subcutaneous nodules, scars, and no enlargement of superficial lymph nodes throughout the body. The lips were free of cyanosis and the oral mucosa was normal. The pharyngeal mucosa is normal, the tonsils are not enlarged, The trachea is in the

S.N.	Site	Screening #	Subject Name	Name	Scenario	Severity	Actions Taken	Outcome	Relationship with the Investigational Device	Details of Occurrence and Treatment
										middle, the breathing movement is normal, the breathing sounds in both lungs are thick, and there is no eminence in the precordial area, Apical pulsation point at left fourth intercostal midclavicular line, apical pulsation normal, uncardiac Bag friction. Test result return: blood routine: white blood cell count (WBC) 7.55 × 10 ^ 9/L; Neutrophil ratio (NE) 0.525 ratio; Lymphocyte ratio (LY) 0.404; Red blood cell count (RBC) 3.85 × 10 ^ 12/L; Hemoglobin (HGB) 113g/L; Total platelet count (PLT) was 207 × 10 ^ 9/L. There were no significant abnormalities in liver and kidney function, myocardial enzymes, electrolytes and blood coagulation. No abnormalities were found in the three-dimensional reconstruction of head CT. Instructions to improve the head-up tilt table test, fat-soluble vitamins, to be treated with calcium gluconate, the rest of the treatment continued to be the same as before, and to observe the changes in the condition of the children. The subject was admitted to the hospital at 18:00 pm on February 15, 2022, and the investigator was informed that the time was February 15, 2022, and the first report of serious adverse events was submitted on February 17, 2022. The reason why the SAE report was reported for more than 24 hours for the first time: After the subject developed headache symptoms, he came to the Department of Cardiovascular Medicine of the hospital to request admission for treatment and investigation, and was received by Dr. Zeng Min. During the hospitalization on June 5, 2021, Dr. Zeng Min did not understand the screening status of the subject after signing the informed consent form, and did not reflect the need to collect and report SAE, so he did not report it in time this time. Chief Physician Chen Zhi, the principal investigator, collected SAE information after learning about the subject's condition when communicating the condition,

S.N.	Site	Screening #	Subject Name	Name	Scenario	Severity	Actions Taken	Outcome	Relationship with the Investigational Device	Details of Occurrence and Treatment
										and submitted the first report of serious adverse events on February 17, 2022.<br>On the evening of February 15, 2022, the subject underwent relevant examinations after admission, blood biochemistry: liver function: total protein (TB) 61.6 g/L ↓; Total bile acids (TBAC) 14.1 umol/L ↑; Renal function: urea nitrogen (BUM) 7.65 mmol/L ↑. Other blood tests: Fat-soluble vitamins: Vitamin A (VA) 778.02 ng/ml; Vitamin E (VE) 12.21 ug/ml; Vitamin K (VK) 0.76 ng/ml; Vitamin D2 (15-0HD2) 15.04 ng/ml; Vitamin D3 (25-0HD3) 13.06 ng/ml; Vitamin D (D2D3) 28ng/ml. On February 16, 2022, he perfected the MRI films from other hospitals and sent them to the Radiology Department of our hospital for reading. The head CT examination in our hospital did not indicate clear infarction, hemorrhage and other manifestations. On February 16, 2022, the head tilt test was negative. Vitamin C injection (Yichang 0.25 Qd) will be given from February 15, 2022 to February 19, 2022; From February 16, 2022 to February 19, 2022, calcium gluconate injection (Linzhou 10%) (10ml Qd); From February 17, 2022 to February 19, 2022, vitamin D drops (capsule 400IU Qd oral), vitamin E soft capsules (Xiamen 100mg Bid oral); From February 18, 2022 to February 19, 2022, aspirin-coated intestinal tablets (100 mg Qd orally) and other symptomatic treatment, no headache occurred after admission, please refer to the Department of Neurology for consultation, it is recommended to improve blood lead, nerve conduction, Cervical spine MRI, blood lipids, immune-related examinations, DSA, and considering that many members of the child's family have a history of cerebral infarction, it is recommended to improve genetic testing if necessary. After the subject was admitted to the hospital, there was no headache attack, the general condition was

S.N.	Site	Screening #	Subject Name	Name	Scenario	Severity	Actions Taken	Outcome	Relationship with the Investigational Device	Details of Occurrence and Treatment
										acceptable, and there was no clear intracranial abnormality. The subject's guardian mother refused blood lead, nerve conduction, cervical spine MRI and other related examinations, and asked to be discharged on 2.19/2022. Diagnosis: 1. Headache tracking; 2. After closure of atrial septal defect; 3. Meniscus injury? Considering that the subject is a swimmer who underwent a high intensity of training from 3 months after surgery, it is recommended to reduce the intensity of training. It has been told to observe the changes of symptoms dynamically and pay close attention.<br>On June 20, 2022, the subjects came to our hospital for a follow-up of 12 months after surgery. Blood routine, urine routine urine sediment, liver and kidney function, blood pregnancy, heart color Doppler ultrasound, electrocardiogram and other examinations were performed. Urine routine urine sediment: white blood cell +-(NCS), white blood cell count 28/ul (NCS), epithelial cell count 122/ul (NCS), bacteria 2/ul (NCS), small round cell count 1/ul (NCS), heterogeneous red blood cell 4/ul (NCS), urobilinogen +-(NCS), liver and kidney function: alanine aminotransferase (ALT) 3.6 IU/L (NCS), urea nitrogen (BUN) 7.28 mmol/L (NCS), the rest Normal blood routine, negative blood pregnancy, heart color Doppler ultrasound: 1. The position of the atrial septal occluder is normal, the range is reduced, the echo is reduced, and there is no obvious blood flow through the septum around. 2. Normal range of left heart systolic function. Electrocardiogram: roughly normal electrocardiogram. After the subject was discharged from the hospital on February 19, 2022, there were no dizziness, intermittent amaurosis, headache, and no other special discomfort. Medication: 1. Aspirin enteric-coated tablets, 2022.2.18-2022.5.26, 100mg, Qd.2, vitamin E soft capsules, 2022.2.17-2022.5.26,

S.N.	Site	Screening #	Subject Name	Name	Scenario	Severity	Actions Taken	Outcome	Relationship with the Investigational Device	Details of Occurrence and Treatment
										100mg, Qd.3, calcium carbonate D3 granules, 2022.2.19-2022.5.26, 0.5 g, Qd.4, complex vitamin B tablets, 2022.2.19-2022.5.26, 1 tablet, Qd.5, coenzyme Q10 capsules, 2022.2.19-2022.5.26, 10mg, Tid.. Filed SAE summary report on June 21, 2022.
**10**	7	0738	ZRNI	1. chronic superficial gastritis;<br>2. Helicobacter pylori infection	Leading to hospitalization	Moderate	Continued in the trial	Resolved without sequelae	Not related	Screening number BASD-07-38 Subject ZRNI was hospitalized on October 11, 2023 and voluntarily signed an informed consent form to participate in the "Biodegradable ASD Occluder and Delivery Device Effectiveness and Safety" sponsored by Shanghai Mallow Medical Devices Co., Ltd. The prospective, multi-center, randomized controlled "clinical trial met the selection criteria of this trial on October 12, 2023, and did not meet the exclusion criteria of this trial, so it was selected for this trial. The subject underwent atrial septal defect occlusion on October 12, 2023. During the operation, the M-DS-8F-850/45 heart defect occluder delivery device and M-ASD-12mm metal single-riveted ASD occluder were used. device, the operation went smoothly. The general condition of the postoperative subjects can be. After discharge, the subjects came to our hospital on November 14, 2023, January 15, 2024, and April 15, 2024 for relevant visits and examinations in January, March, and June after surgery. AE events and SAE events related to the test device occurred. The father of the subject's legal guardian consulted the researcher of this clinical center by phone at 16:30 pm on July 31, 2024, and informed the researcher that the subject came to the Department of Gastroenterology of our hospital for treatment due to abdominal pain on July 25, 2024., admitted to the hospital for treatment. The Department of Digestive Nutrition learned that the subject began to develop paroxysmal abdominal pain 10

S.N.	Site	Screening #	Subject Name	Name	Scenario	Severity	Actions Taken	Outcome	Relationship with the Investigational Device	Details of Occurrence and Treatment
										months ago, predominantly under the xiphoid process and around the umbilical cord, aggravated the pain after eating, which can be relieved spontaneously, without nausea and vomiting, and without diarrhea, and did not pay attention to it. When the subject participated in this clinical trial 9 months ago, he was in good health, and the above-mentioned situation did not occur, and no abnormalities were found in the relevant examinations during the screening period. After the operation and before discharge, he was hospitalized for one day due to fever and reported to the ethical SAE. According to the analysis of the researcher, it was considered that it was caused by bacterial infection and had nothing to do with the test device. The SAE incident was reported to the SAE summary report during the follow-up period of 1 month after the operation. After eating milk 2 days ago (July 23, 2024), the subjects had aggravated abdominal pain and vomited once, which was stomach content, no blood or bile was seen, and the pain could not be relieved after vomiting. The child has no fever, abdominal distension, diarrhea, and no blood in the stool. Since the onset of the subject, the mental appetite is not good, and the sleep can be good. On July 25, 2024, I came to Hunan Children's Hospital for abdominal color Doppler ultrasound and intestinal color Doppler ultrasound: abdominal lymph nodes were enlarged. Abdominal flatulence. The outpatient department is admitted to the hospital, and the admission diagnosis is: 1. Abdominal pain 1.1. Chronic gastritis (?) 1. 2IgA vasculitis) 1. 3 Acute pancreatitis). After admission, improve various examinations: three routine, thoracoabdominal orthostatic film, electrolytes, liver and kidney function, c13 breath test, blood and urine amylase, blood lipase, abdominal and intestinal color Doppler ultrasound, etc., and improve gastrointestinal

S.N.	Site	Screening #	Subject Name	Name	Scenario	Severity	Actions Taken	Outcome	Relationship with the Investigational Device	Details of Occurrence and Treatment
										endoscopy at an optional date Assist in diagnosis and treatment. Esomeprazole powder injection QD1010mg intravenous infusion for acid suppression and stomach protection, vitamin B6 injection QD10100mg, sodium bicarbonate injection, 5% glucose injection, 10% potassium chloride, etc. were given for symptomatic and supportive treatment. Painless gastroscopy was performed on July 26, 2024. Occasional abdominal pain, no nausea and vomiting, no diarrhea and blood in the stool, good spirit, and normal urination. Results: Erythrocyte sedimentation rate (ESR), blood routine plus whole blood CRP, coagulation set, four items of blood transfusion, and urine routine (urinalysis) were not abnormal. Nine items of liver function + kidney function + blood glucose (GLU): glucose (GLU) 2.88 mmol. Checking thoracic and abdominal orthostatic type photography: 1. Changes after closure of atrial septal defect. 2. No obvious signs of obstruction were found in the abdomen. Checking electronic gastroscopy/duodenoscopy + endoscopic staining: chronic superficial gastritis. 13C breath test Hp test report results: positive. Electrocardiogram: 1. Sinus rhythm 2. Incomplete right bundle branch block. The diagnosis of the child is currently considered: 1. Chronic superficial gastritis 2. Helicobacter pylori infection, given phloroglucinol injection 20mg ONCE, aluminum magnesium carbonate chewable tablets 0.5 g TID, amoxicillin potassium clavulanate 15mg/kg q12 hours for 14 days, esomeprazole powder injection 10mg/QD for 10 days, clarithromycin 125mg/time q12h course for 14 days of anti-Helicobacter pylori infection treatment. On July 27, 2024, the subject occasionally had abdominal pain, which was better than before, no diarrhea and blood in the stool, good mental appetite, and normal defecation. Results: Antistreptolysin (ASO)

S.N.	Site	Screening #	Subject Name	Name	Scenario	Severity	Actions Taken	Outcome	Relationship with the Investigational Device	Details of Occurrence and Treatment
										+ immunization had no abnormalities, and 11 items of allergen IgE were checked: egg (egg) 0.48 (grade 1) IU/mL, milk (milk) 0.19 IU/mL. Anti-Helicobacter pylori therapy was continued. On July 29, 2024, the subject had no abdominal pain, abdominal distension, no nausea and vomiting, no blood in the stool, good mental appetite, and normal defecation. Results There were no abnormalities in the OB test. Continuing treatment against Helicobacter pylori infection. On July 30, 2024, the subject had no abdominal pain, vomiting, and was able to eat mentally. The current condition is better than before. He was discharged from the hospital and brought medicine: 1. Omeprazole enteric-coated tablets 10mg/time once a day (morning take) (total course of treatment for gastric ulcer 6-8 weeks, bring yourself); 2. Amoxicillin potassium clavulanate 390mg/time 2 times a day oral total course of treatment for 14 days (self-provided); 3. Clarithromycin 125mg/time, 2 oral doses per day, total course of treatment for 14 days (self-provided); 4. Aluminum magnesium carbonate tablets 0.5 g orally 3 times a day after meals (bring yourself). It is recommended to re-visit the outpatient department of digestive nutrition half a month later, re-examine the electronic gastroscope if necessary, and re-examine the 13C breath test after one month of Helicobacter pylori positive withdrawal; Light, digestible soft food, eat regularly and quantitatively, do not eat spicy, irritating snacks and carbonated drinks and other snacks; Seek a doctor in time when diarrhea, abdominal pain, fever, etc. occur. If you feel unwell, see a doctor in time. The researcher learned of the SAE incident from the subject's legal guardian this time, checked the hospitalization course system, read and analyzed the relevant examinations of the subject during hospitalization, and determined that the SAE incident

S.N.	Site	Screening #	Subject Name	Name	Scenario	Severity	Actions Taken	Outcome	Relationship with the Investigational Device	Details of Occurrence and Treatment
										must have nothing to do with the test device and will not affect the subject. After the occluder is implanted, it will bring related device complications to the body that may be caused by such devices. The first report of serious adverse events will be submitted on August 1, 2024, and follow-up will be paid close attention to. The researcher called the father of the subject's legal guardian at 09:58 on August 12, 2024 to learn about the subject's recovery after discharge. The subject's legal guardian complained that "the subject currently has no abdominal distension, abdominal pain, nausea and vomiting, diarrhea, and blood in the stool. Waiting for mental intake. Discharge medication status: 1. Omeprazole magnesium enteric-coated tablets 10mg/time once a day Start and end time: July 30, 2024-August 9, 2024; 2. Amoxicillin clavulanate potassium dry suspension 390mg/time twice a day Start and end time: July 30, 2024-August 9, 2024; 3. Clarithromycin dispersible tablets 125mg/time twice a day Start and end time: July 26, 2024-August 9, 2024 The subject came to the hospital on October 14, 2024 for a long-term observation and follow-up of 12 months after the operation. The general situation is good. The examination of this clinical item shows that the liver and kidney functions are normal. Blood routine: Blood routine: Leukocyte 4.19 x10 ^ 9/L (NCS), neutrophil number 1.13 X10 ^ 9/L (NCS), rest normal. Urine routine + urine sediment: white blood cells +-(NCS), red blood cell count 9/u (NCS), white blood cell count 16/uL (NCS), the rest were normal. Cardiac color Doppler ultrasound: 12 months after atrial septal defect closure (metal type): 1. There is no obvious blood color around the atrial septal occluder. 2. Normal range of left heart systolic function. Electrocardiogram: 1. Sinus rhythm. 2. Incomplete right bundle branch block? (NCS). Re-examination by the Department of Digestion

S.N.	Site	Screening #	Subject Name	Name	Scenario	Severity	Actions Taken	Outcome	Relationship with the Investigational Device	Details of Occurrence and Treatment
										and Nutrition of SAE events: tenderness in the upper abdomen around the umbilical cord, liver and spleen (I), and normal bowel sounds. 13-carbon urea breath test: positive, regular re-examination. The condition of the subject has improved, and there is no need for further relevant treatment and disposal, and the condition is stable. The SAE summary report is now submitted on October 15, 2024.
**11**	13	1301	HJLI	Abnormal uterine bleeding	Leading to hospitalization	Mild	Continued in the trial	Resolved without sequelae	Not related	Patient HJLI, female, 34 years old, Han nationality, signed an informed consent form on September 15, 2022, expressing his willingness to participate in the "Prospective, Multicenter, Randomized Controlled Clinical Trial on the Efficacy and Safety of Degradable ASD Occluders and Delivery Devices" (protocol number: JK-LC-BASD-01), and then obtained the screening number BASD-13-01, the researcher judged that it met the selection criteria for this trial based on the examination results and did not meet any of the exclusion criteria, and was enrolled in the clinical trial of degradable ASD occluder on September 16, 2022. On September 16, 2022, the "Congenital Heart Disease Atrial Septal Defect Closure Surgery" was performed. The patient has completed a 12-month follow-up after ASD closure on September 5, 2023. The researcher learned on May 28, 2024 that the subject had suffered from irregular vaginal bleeding for one year, and was admitted to the gynecology department of our hospital for hospitalization on July 11, 2023. After admission, the relevant examinations were perfected. Blood routine analysis: hemoglobin: 112.0 g/L; Single disease liver and kidney glucose electrolyte: alanine aminotransferase: 111U/L; Aspartate aminotransferase: 86U/L; Four examinations for infectious diseases: hepatitis B surface antigen: > 250.00 IU/mL. No significant abnormalities were found in three items of

S.N.	Site	Screening #	Subject Name	Name	Scenario	Severity	Actions Taken	Outcome	Relationship with the Investigational Device	Details of Occurrence and Treatment
										thyroid function, urine routine, gynecological tumor markers, and electrocardiogram. Electrocardiographic tip: sinus bradycardia. On July 12, 2023, under general anesthesia, hysteroscopic diagnostic curettage, hysteroscopy, hysteroscopic uterine lesion resection (cervical radiofrequency ablation), and intrauterine contraceptive device placement, the operation went smoothly, and the postoperative pathological examination results were not returned. Postoperative treatment such as prevention of infection, rehydration, stomach protection and hemostasis were given. Postoperative reexamination; Blood routine analysis: hemoglobin: 102.0 g/L; Coagulation + dimer: prothrombin time: 12.8 s; Prothrombin normalized ratio: 1.17. Now the general condition of the patient is good, with a small amount of vaginal bleeding, no abdominal pain and abdominal distension. The patient was discharged from the hospital on July 13, 2023, and the SAE will end on July 13, 2023. Today, the first summary report of SAE "abnormal uterine bleeding" is reported. It is judged to be related to the experimental medical device Definitely irrelevant.
