# Supplementary material for: A scaffold-degradable single-rivet ASD occluder with amplatzer-like design: a multicenter randomized controlled trial
Source: Cardiovasc Interv Ther. 2026 Jun 19;41(4):1161–72. doi: 10.1007/s12928-026-01299-7 (PMC13582343; doi:10.1007/s12928-026-01299-7)
Supplement: Supplementary file 8 [file 12928_2026_1299_MOESM8_ESM.pdf]

The Affiliated Tumor Hospital of Fudan University
Pathology consultation opinion of other hospital pathology section

Undergraduate consultation number: t2023-18211

T2023-18211

Patient's name: Xie Xiaorong   Gender: Female Age: 8 years old   Date of receipt: 2023-08-08

Contact address and communication information: 15921710307

Inspection institution: Xinhua Hospital

Slide number: p202303027 he: 3 pieces; white film: 15 pieces

Pathology consultation opinion:

Submit 3 he and corresponding white slides for examination, after re-staining:

Immunohistochemistry results (hi23-23859) 3 sets of white slides

C034 (VASCULAR ENDOTHELIAL CELLS +), SMA (FIBROBLASTS +), LCA (LYMPHOCYTES +)

Initial doctor: Ni Shujuan   Follow-up doctor: Ni Shujuan

(Signature is valid)

Note: The pathologist's personal consultation opinion is for reference only by the pathologist who made the original pathological diagnosis. Report date: 2023-08-17

Address: No. 270 Dong'an Road, Shanghai, Postal Code: 200032

Xinhua Hospital Affiliated to Shanghai Jiaotong University School of Medicine

Pathology diagnosis report

P202303027

Pathology Number: P202303027

Department: Pediatric Operating Room Received date: 2023-07-05

Name: Xie Xiaorong   Gender: Female   Age: 8

Clinic number: 79720860   Hospital number:L16233   Ward: Children's Heart Center Monitoring Bed   Number: 06

Gross findings: Atrial septum and occluder: A piece of gray-yellow tissue, 0.6 cm in diameter.

Pathological diagnosis: Foreign bodies with multinucleated giant cell reaction, fibrosis and granulation tissue hyperplasia, scattered chronic inflammatory cell infiltration, and no other obvious abnormalities were found. Please combine with clini cal findings.

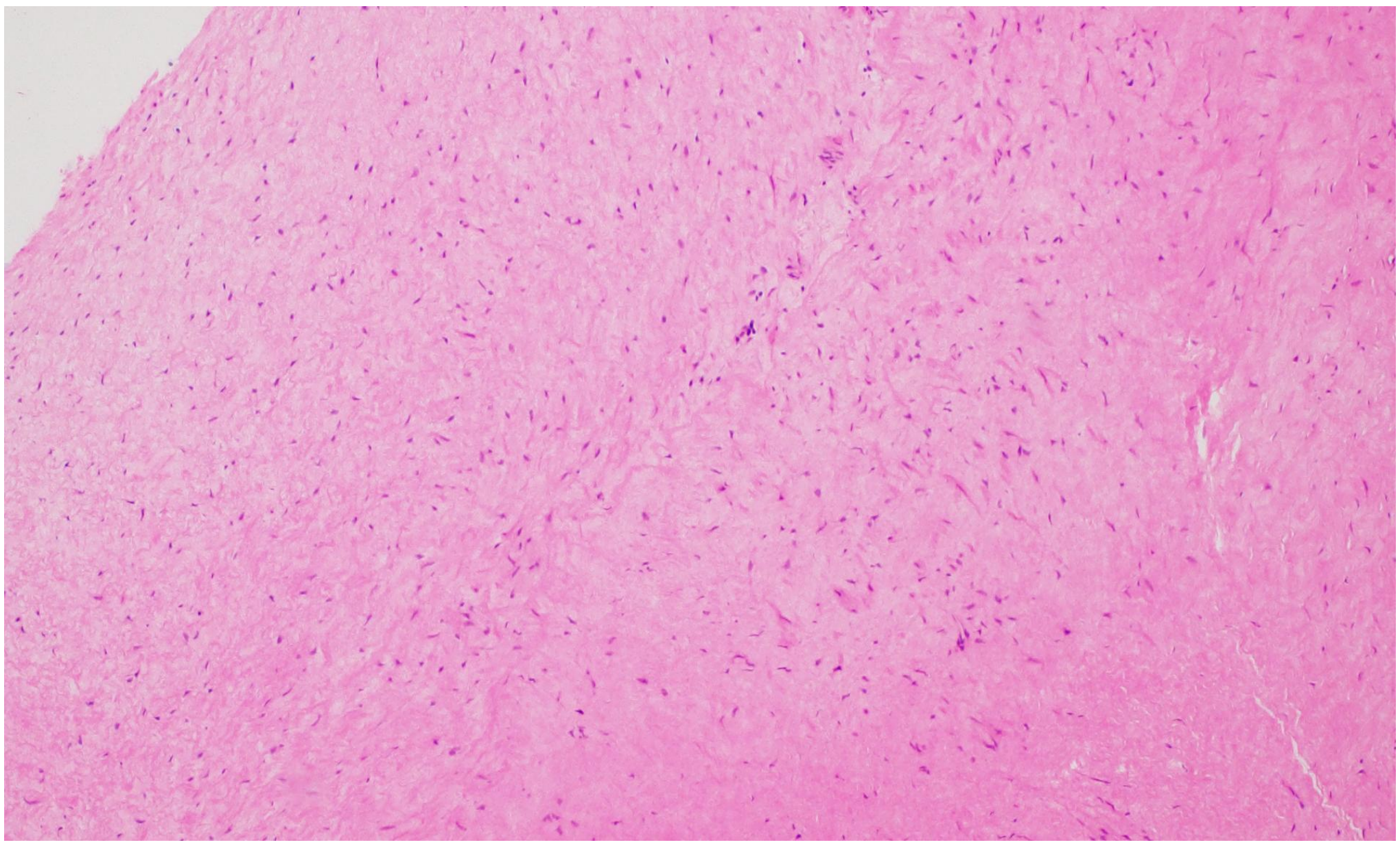

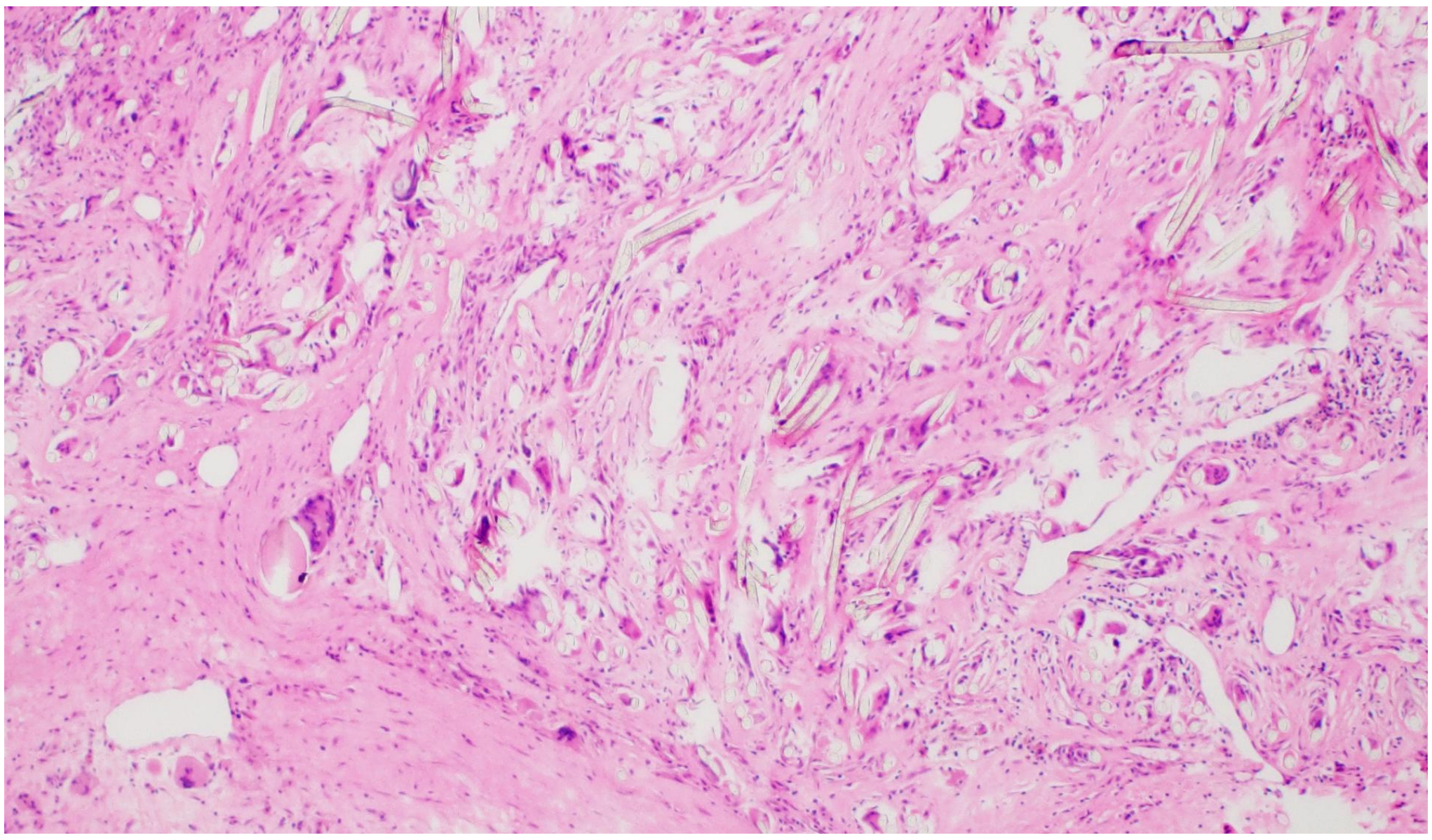

First visit physician : Liang Chunwu   Diagnostic physician: Li Honghua   Report date: 2023-07-10

＊＊ If the above diagnosis is inconsistent with the clinical findings, please contact our department in time

Address: Pathology Department, 4th Floor, Medical Technology Building, No. 1665, Kongjiang Road, Shanglei City
